# Ferroptosis in human reproductive tract infections and associated disorders: mechanisms and emerging therapeutic opportunities

**DOI:** 10.3389/fimmu.2025.1713074

**Published:** 2025-12-17

**Authors:** Xinchen Wan, Xue Wang, Yanjun Liu, Longyan Hong, Zhandi Zhao, Pengbo Guo

**Affiliations:** 1 Department of Clinical Laboratory Medicine, The First Affiliated Hospital of Shandong First Medical University and Shandong Provincial Qianfoshan Hospital, Shandong Medicine and Health Key Laboratory of Laboratory Medicine, Jinan, Shandong, China; 2 School of Clinical and Basic Medicine, Shandong First Medical University and Shandong Academy of Medical Sciences, Jinan, Shandong, China; 3 Department of Gynecology, Juxian People’s Hospital, Rizhao, Shandong, China

**Keywords:** ferroptosis, HIV, HPV, molecular mechanism, SARS-CoV-2, *Staphylococcus aureus*, therapeutic target

## Abstract

Ferroptosis, an iron-dependent form of regulated cell death driven by lipid peroxidation, is closely associated with mitochondrial damage, diminished glutathione peroxidase 4 activity, dysfunction of the System Xc^−^ cystine/glutamate antiporter, and disruptions in iron metabolism. Infections of the human reproductive system and associated reproductive disorders pose a significant global public health challenge, characterized by diverse pathogens and complex pathogenic mechanisms. Recent research has revealed that ferroptosis plays a critical role in the pathological processes of many of these infections. This review systematically elaborates on the central mechanistic role of ferroptosis in various pathologies of the reproductive system. These include CD4^+^ T cell depletion and immunological non-response in Human Immunodeficiency Virus (HIV) infection, the development of Human Papillomavirus (HPV)-associated cervical cancer, *Staphylococcus aureus*-induced endometritis and mastitis, as well as male infertility, pre-eclampsia, and ovarian cancer linked to Severe Acute Respiratory Syndrome Coronavirus 2 (SARS-CoV-2) infection. Despite the diversity of the pathogens, they can all trigger ferroptosis through common mechanisms, such as disrupting the Nrf2/GPX4 antioxidant axis, impairing the System Xc^−^–GSH–GPX4 pathway, and inducing dysregulation of iron metabolism. Furthermore, ferroptosis interacts intricately with pyroptosis and apoptosis, forming a complex network that collectively regulates the outcome of infections and the extent of tissue damage. Notably, ferroptosis plays a context-dependent dual role in various reproductive system infections. During the initial phases of infection, it exerts a protective effect by eliminating pathogens and curbing infection progression. In contrast, during advanced or chronic stages, ferroptosis exacerbates tissue injury and promotes disease pathogenesis. The ferroptosis pathway holds great therapeutic promise, either through inhibitors that safeguard host cells or inducers that eradicate drug-resistant bacteria by triggering a “ferroptosis-like” state. Nevertheless, challenges remain for clinical translation, as the ferroptosis network is incompletely understood, and the tissue selectivity and long-term safety of targeted drugs are unverified. Future studies must elucidate host-pathogen interactions to develop precise targeted therapies.

## 1 Introduction

Human reproductive tract infections and associated disorders refer to a group of disorders resulting from the colonization and invasion of urogenital organs and associated reproductive tissues, including the uterus, ovaries, fallopian tubes, vagina, penis, testes, epididymis, and mammary glands, by pathogenic microorganisms such as bacteria, viruses, fungi, and parasites. These infections are primarily transmitted through sexual contact or vertical transmission from mother to child. Human reproductive tract infections and associated disorders elicit inflammatory reactions that may be localized or systemic, leading to tissue dysfunction and eliciting a range of clinical manifestations. RTIs represent major causative factors in various reproductive health complications, including infertility, spontaneous abortion, preterm delivery, and neonatal infections. Owing to their diverse etiological agents, varied transmission routes, and heterogeneous clinical presentations, Human reproductive tract infections and associated disorders constitute a substantial challenge in global reproductive medicine and public health. Despite the diverse clinical manifestations and pathogens involved in human reproductive tract infections and related disorders, recent studies have identified ferroptosis—an iron-dependent form of regulated cell death—as playing a critical role in the pathogenesis of many such infections, serving as a common molecular bridge connecting pathogen infection to host tissue damage.

Ferroptosis is a distinct form of regulated cell death, mechanistically independent of autophagy, apoptosis, and necrosis. Its initiation is primarily driven by intracellular iron dyshomeostasis, excessive accumulation of lipid peroxides, and failure of the antioxidant defense system. Morphologically, ferroptosis is characterized by specific ultrastructural alterations, most notably mitochondrial shrinkage, reduction or loss of cristae and increased mitochondrial membrane density (condensation). In contrast, the plasma membrane and nuclear envelope remain intact, and no apoptotic bodies are formed. The core pathological mechanism involves iron-dependent and dysregulated accumulation of lipid peroxidation (1). The key negative regulator of ferroptosis is glutathione peroxidase 4 (GPX4). Inhibition of System Xc^−^ function reduces cellular cystine uptake, leading to depleted glutathione (GSH) synthesis, subsequent loss of GPX4 activity, and ultimately a failure of the cellular antioxidant system (2). Disrupted iron homeostasis further contributes to ferroptotic cell death. Intracellular iron overload catalyzes Fenton reactions, resulting in a surge of reactive oxygen species (ROS) that promote lipid peroxidation and exacerbate oxidative stress cascades. The progressive accumulation of lipid peroxides inflicts systemic damage on cellular membranes, compromising membrane integrity and ultimately executing cell death (3, 4).

Emerging evidence highlights the significant role of ferroptosis in reproductive tract pathologies induced by various pathogens. Ferroptosis contributes to CD4^+^ T cell depletion in Human Immunodeficiency Virus (HIV) infection. In Human Papillomavirus (HPV) infection, ferroptosis is implicated in cervical carcinogenesis. Ferroptosis exacerbates *Staphylococcus aureus* (*S. aureus*)-induced endometritis and mastitis. Furthermore, during SARS-CoV-2 infection, ferroptosis induces significant damage to the reproductive system in the context of ACE2 receptor-mediated multiorgan invasion.

Targeting ferroptosis pathways offers significant therapeutic potential for managing these pathogen-associated reproductive infections. This review systematically elucidates the roles, molecular mechanisms, and therapeutic strategies related to ferroptosis in the context of these infections. Our analysis aims to advance the development of novel treatment strategies and provide new interventions against drug-resistant bacterial infections.

## 2 Ferroptosis and human reproductive tract infections

### 2.1 HIV

HIV is an enveloped, single-stranded RNA retrovirus. It is transmitted primarily through sexual contact, exposure to contaminated blood products, and vertical transmission from mother to child. A hallmark of HIV pathogenesis is its specific tropism for and destruction of the host immune system, particularly CD4^+^ T lymphocytes, which serve as the primary cellular reservoir for the virus. During the late stages of infection, HIV induces massive depletion of CD4^+^ T lymphocytes, leading to acquired immune-deficiency syndrome (AIDS). This condition is defined by severe immunosuppression, which predisposes patients to life-threatening opportunistic infections and malignancies.

Highly Active Antiretroviral Therapy (HAART) constitutes the current standard of care for HIV infection. Effective HAART suppresses viral replication and facilitates the reconstitution of CD4^+^ T cell counts. However, approximately 10% – 40% of patients exhibit a paradoxical immunological phenomenon: despite achieving sustained virological suppression (plasma HIV RNA <50 copies/mL), CD4^+^ T cell counts persistently remain below 200 cells/μL. These individuals are classified as Immunological Non-Responders (INRs) (5). Compared to immunological responders, INRs face significantly elevated risks of opportunistic infections, higher incidence of malignancies, and increased all-cause mortality.

#### 2.1.1 CD4^+^ T cells ferroptosis and INRs

Recent studies demonstrate that compared to partial responders (PRs) and complete responders (CRs), CD4^+^ T cells from INRs exhibit more pronounced ferroptotic alterations. These characteristics include mitochondrial dysfunction, metabolic dysregulation and significant downregulation of GPX4 expression. Furthermore, ferroptosis critically impairs key CD4^+^ T cell function such as suppression of cellular proliferation and attenuation of activation capacity. This ultimately hinders immune reconstitution following HAART.

Mounting evidence demonstrates a pivotal association between CD4+ T cell ferroptosis and the immunological impairment observed in INRs.

**Figure 1:**
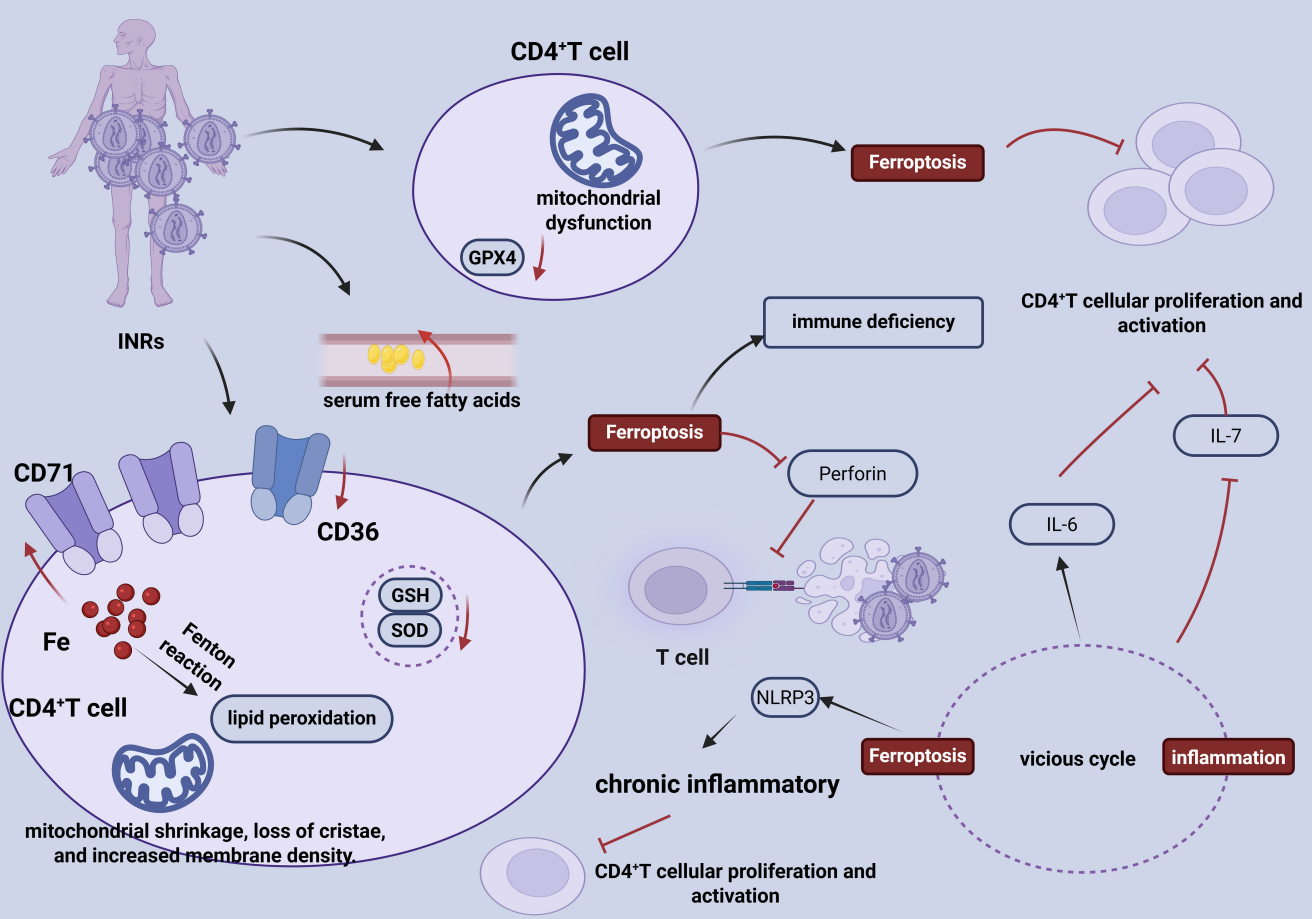
Ferroptosis and HIV infection correlation schematic diagram. In patients with INRs, CD4^+^ T cells exhibit characteristic features of ferroptosis, including downregulated expression of GPX4 and mitochondrial dysfunction. This process is driven by multiple factors: elevated serum free fatty acid levels, altered expression of the transferrin receptor (CD71) on the cell membrane, upregulation of the lipid transporter CD36, abnormal mitochondrial structure, and intracellular iron accumulation, collectively promoting lipid peroxidation. Concurrently, reduced levels of antioxidants such as GSH and SOD further exacerbate the ferroptosis process. Ferroptosis not only exacerbates the immunodeficiency state in INR patients but also reduces intracellular perforin levels, thereby impairing T cell-mediated pathogen clearance. Furthermore, ferroptosis and inflammatory responses form a vicious cycle: on one hand, it inhibits IL-7-induced CD4^+^ T cell proliferation, and on the other hand, it enhances the suppressive effect of IL-6 on T cell response to IL-7. Ferroptosis can also activate the NLRP3 inflammasome, leading to a chronic inflammatory state and further suppressing the activation and function of CD4^+^ T cells.

Firstly, mitochondrial dysfunction, recognized as early hallmarks of ferroptosis, is observed in CD4^+^ T cells from INRs patients. Studies have revealed a significant reduction in mitochondrial membrane potential, indicating depolarization consistent with initial ferroptotic damage. Further corroboration comes from transmission electron microscopy, which demonstrates characteristic ultrastructural alterations including mitochondrial shrinkage, loss of cristae, and increased membrane density (**Figure 1**) (6).

Secondly, CD4^+^ T cells from INRs exhibit elevated free iron ions and a pronounced accumulation of lipid peroxidation, representing the core drivers of ferroptosis. These changes are accompanied by increased serum free fatty acids, significant upregulation of the transferrin receptor CD71, downregulation of the fatty acid transporter CD36, and substantially reduced intracellular levels of antioxidant substances GSH and superoxide dismutase (SOD). The accumulation of intracellular iron catalyzes Fenton reactions, generating excessive ROS that initiate widespread lipid peroxidation. This cascade enhances the susceptibility of CD4^+^ T cells to ferroptosis, thereby exacerbating immune deficiency (**Figure 1**) (6).

Thirdly, cytotoxic dysfunction is evident in INR patients. Perforin levels in CD4^+^ T cells, which are negatively correlated with ferroptosis markers, are significantly reduced, l impairing the antiviral activity of cytotoxic T lymphocytes (**Figure 1**) (6).

Finally, a vicious cycle of inflammation and ferroptosis sustains CD4^+^ T cell deficiency in INRs. The capacity of interleukin-7 (IL-7) to induce CD4^+^ T cell proliferation is impaired. Elevated plasma interleukin-6 (IL-6) levels further suppress CD4^+^ T cell responsiveness to IL-7, resulting in inhibited proliferation (5). Simultaneously, ferroptosis-related peroxides persistently activation of the NLRP3 inflammasome, fostering a chronic inflammatory microenvironment that continuously suppresses CD4^+^ T cell reconstitution (**Figure 1**) (7).

HIV induces ferroptosis in CD4^+^ T cells through mechanisms involving viral protein-mediated disruption of the host cell’s antioxidant system and dysregulation of lipid peroxide accumulation. The specific mechanisms encompass three key aspects. First, the HIV-1-encoded Tat protein promotes ferroptosis via the miR-204-ACSL4 signaling axis. Tat significantly downregulates intracellular miR-204 expression, thereby relieving its suppression of the target gene ACSL4. The subsequent upregulation of ACSL4 enhances the lipid peroxidation of polyunsaturated fatty acids, ultimately inducing ferroptosis (8). Second, the Tat protein interferes with selenium metabolism in T cells, leading to reduced expression of key antioxidant selenoproteins, including GPX4, and weakening the cellular antioxidant defense system (9). As GPX4 is a crucial negative regulator of ferroptosis, its decreased expression facilitates the occurrence of ferroptosis. Third, the HIV capsid protein gp120 contributes to iron metabolism dysregulation by disrupting endolysosomal function. Following its internalization into endolysosomes, gp120 causes an increase in endolysosomal pH. This promotes the release of stored iron ions into the cytosol through the TRPML1 channel, resulting in iron overload in both the cytosol and mitochondria. The excess iron ions then catalyze the generation of lipid peroxides via the Fenton reaction, further driving the ferroptosis process (10).

Collectively, these mechanisms demonstrate that HIV proteins directly regulate core ferroptosis pathways, establishing a direct causal link from viral proteins to ferroptosis in host cells.

#### 2.1.2 Targeting ferroptosis for HIV treatment: potential and challenges

Ferroptosis plays a pivotal role in HIV infection and the impairment of immune reconstitution. Targeting ferroptosis regulation demonstrates significant therapeutic potential for managing HIV infection and improving clinical outcomes.

Firstly, inhibition of ferroptosis in INRs can attenuate aberrant CD4^+^ T cell death, thereby preserving this essential lymphocyte population. Secondly, ferroptosis suppression could help mitigate chronic inflammation, subsequently reducing immune activation and exhaustion of CD4^+^ T cells. Thirdly, given the role of chronic inflammation in promoting viral proliferation and replication, suppressing ferroptosis and its associated inflammatory microenvironment may contribute to a less favorable environment for the virus, potentially suppressing activity within the HIV reservoir and further curbing viral proliferation (11). Lastly, inhibiting HIV-associated ferroptosis in intestinal epithelial cells can effectively mitigate gut barrier damage and reduce systemic inflammation resulting from microbial translocation (12).

Experimental studies have indicated that ferroptosis inhibitors can partially restore mitochondrial function, attenuate inflammatory responses, and reverse metabolic dysregulation in INRs (6). Consequently, a deeper understanding of the mechanisms linking HIV infection and CD4^+^ T cell ferroptosis will be essential for developing novel therapeutic strategies aimed at controlling viral activity and facilitating immune recovery in INRs. Despite the considerable therapeutic potential of targeting ferroptosis for HIV infection and immune recovery in INRs, its clinical application faces multiple challenges. First, the molecular mechanisms through which HIV infection induces ferroptosis are not yet fully elucidated, and the crosstalk between ferroptosis and other forms of programmed cell death in HIV infection is still unclear. Second, the complex interaction network between ferroptosis and inflammatory immune responses has not been fully deciphered. Clarifying the role of ferroptosis within the chronic inflammatory microenvironment in HIV infection is crucial. Finally, further investigation is needed to unravel the specific mechanisms by which ferroptosis modulates HIV viral replication and reservoir activation. A deeper understanding of how ferroptosis influences viral replication and proliferation is essential for developing targeted therapeutic strategies.

Future research should aim to elucidate the specific mechanisms of the HIV-host interaction, systematically clarifying how HIV infection induces cellular ferroptosis and how ferroptosis in turn impacts HIV viral replication and immune regulation. In parallel, efforts must be directed toward exploring novel pharmacological agents that target ferroptosis pathways and investigating the therapeutic mechanisms of action against HIV infection.

### 2.2 HPV

HPV, a member of the genus Papillomavirus within the family Papillomaviridae, primarily infects the skin and mucosal epithelial cells of humans, leading to proliferative lesions in epithelial tissues. As a key pathogen associated with diseases of the female reproductive system, HPV is predominantly transmitted through sexual contact. Based on carcinogenic risk, HPV genotypes are categorized into high-risk types (e.g., HPV-16, HPV-18) and low-risk (e.g., HPV-6, HPV-11) types. Infection with low-risk HPV types mainly causes benign lesions such as genital warts and papillomas. In contrast, persistent infection with high-risk HPV types is the major driver of malignant tumors, including cervical cancer. Cervical cancer is the fourth most common malignancy among women globally. According to the WHO, it causes approximately 300,000 deaths in women annually, with a disproportionately high disease burden among middle-aged women and those living in resource-limited settings (13). Current treatment of HPV infection involves antiviral drugs such as ribavirin and acyclovir. For cervical cancer, the main treatment approaches are surgical resection, radiotherapy, and chemotherapy. However, both surgical and chemoradiotherapy treatments are often associated with serious postoperative or treatment-related adverse effects.

#### 2.2.1 Ferroptosis dynamics in cervical carcinogenesis

**Figure 2:**
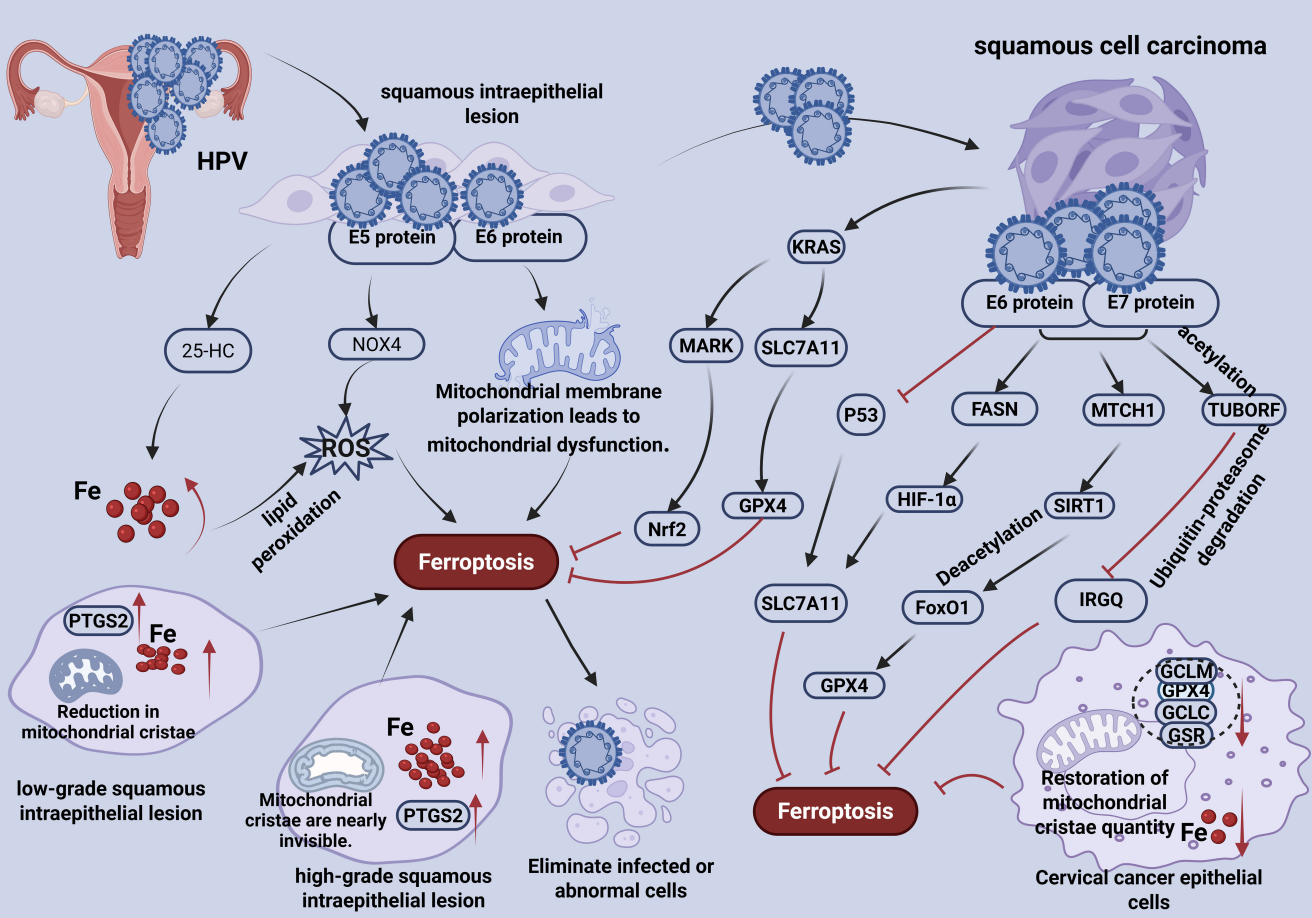
Schematic diagram of the correlation between ferroptosis and HPV-induced SIL and SCC. In cervical SIL, including both LSIL and HSIL stages, HPV infection can promote the occurrence of ferroptosis. SIL cells exhibit characteristic features of ferroptosis, including upregulation of PTGS2 expression, reduction in mitochondrial cristae, and elevated intracellular iron levels, which are particularly prominent in HSIL. Mechanistically, this involves increased intracellular 25-HC content leading to iron accumulation, upregulation of NOX4 expression by the HPV E5 protein resulting in ROS buildup, and mitochondrial dysfunction induced by the E6 protein. SCC cells exhibit anti-ferroptotic properties, characterized by upregulation of antioxidant-related genes such as GCLM, GCLC, GSR, and GPX4, partial restoration of mitochondrial cristae, and lower iron levels compared to the SIL stage. This transition is mediated by multiple pathways: the E6 protein upregulates SLC7A11 through p53 degradation; E6/E7 activate the FASN/HIF-1α/SLC7A11 and MTCH1/SIRT1/FoxO1/GPX4 signaling axes; E6/E7 also promote the expression and acetylation of the TUBORF peptide, leading to IRGQ degradation via the ubiquitin-proteasome system. Additionally, constitutively expressed KRAS enhances the antioxidant response by activating the SLC7A11/GPX4 and MAPK/Nrf2 pathways, further strengthening the anti-ferroptotic capacity in SCC.

The progression from HPV infection to cervical cancer advances through a series of stages, beginning with benign squamous epithelial hyperplasia, followed by low-grade squamous intraepithelial lesion (LSIL), high-grade squamous intraepithelial lesion (HSIL), and ultimately culminating in squamous cell carcinoma (SCC) (14). Recent research has demonstrated that ferroptosis plays a critical role in cervical carcinogenesis induced by high-risk HPV infection. Ferroptotic activity has been detected in premalignant stages, such as endometrial and intraepithelial neoplasia cells, whereas cervical carcinoma cells exhibit significant resistance to ferroptosis (15). These findings suggest a close association between ferroptosis and the malignant transition from intraepithelial neoplasia to cervical carcinoma. Understanding this mechanism may offer a novel therapeutic strategy for controlling HPV infection and treating cervical cancer. Cervical epithelial cells exhibit increased susceptibility to ferroptosis during the SIL stage. Accumulating evidence demonstrates that both LSIL and HSIL cervical lesions exhibit distinct features of ferroptosis. These include elevated expression of the ferroptosis marker PTGS2, abnormal mitochondrial structures characterized by reduced mitochondrial cristae in LSIL and nearly complete loss of cristae in HSIL; increased intracellular iron levels that peak during HSIL, and downregulation of key anti-ferroptosis genes (**Figure 2**). Together, these observations confirm that ferroptosis is the predominant form of cell death during SIL progression (15).

The underlying mechanisms driving ferroptosis in this context involve multiple pathways. Firstly, E5 oncoprotein upregulates the NOX family, continuously generating ROS (16).Secondly, 25-hydroxycholesterol (25-HC) mediates iron homeostasis dysregulation. HPV infection upregulates cellular 25-HC expression, leading to increased interstitial Fe²^+^ concentration, which accelerates lipid peroxidation and enhances ferroptosis sensitivity in HSIL cells (17). Third, E6 protein induces mitochondrial dysfunction by causing mitochondrial membrane depolarization and functional disruption, further promoting ferroptosis (**Figure 2**) (18).

During the SIL stage, ferroptosis serves as a crucial defensive role through a dual mechanism. On one hand, intracellular iron accumulation promotes the Fenton reaction, generating hydroxyl radicals that induce lipid peroxidation, cell membrane rupture, and ultimately ferroptotic cell death, thereby eliminating pre-cancerous or HPV-infected cells. On the other hand, ferroptosis helps release pro-inflammatory factors, which recruit macrophages and other immune cells to enhance immune clearance of HPV and infected cells (**Figure 2**) (19).

During the SCC stage, cervical cancer epithelial cells acquire pronounced anti-ferroptotic capabilities SCC cells exhibit upregulated expression of anti-ferroptosis genes, including GCLM, GCLC, GSR and GPX4. Intracellular iron levels are reduced significantly compared to those in the HSIL stage, and mitochondrial ultrastructure shows recovery of cristae abundance (**Figure 2**) (15). These observations collectively indicate a reversal of ferroptosis or the emergence of ferroptosis resistance in cervical SCC cells.

This adaptive response is mediated through several molecular mechanisms. First, the inactivation of the p53 signaling pathway plays a central role. HPV-derived E6 and E7 oncoproteins promote the degradation of p53/pRB protein. As a transcription factor, p53 can inhibit SLC7A11 transcriptional inhibitors, and its degradation relieves the inhibition of SLC7A11 and enhances the antioxidant capacity of cells (20). Second, antioxidant pathways are upregulated. E6 and E7 increased the expression of fatty acid synthase (FASN), which activates HIF-1α transcription factor, leading to further upregulation of SLC7A11 and reinforcing cellular antioxidant defenses (21). Third, activation of NAD^+^-SIRT1-FoxO1 axis contributes to ferroptosis resistance. E6 and E7 oncoproteins elevate the expression of Mitochondrial Carrier 1 (MTCH1), facilitating NAD^+^ import into mitochondria. Elevated NAD^+^ enhances SIRT1-mediated deacetylation of FoxO1 Deacetylated. FoxO1 binds to the GPX*4* gene promoter, stimulating its expression and increasing the antioxidant capacity, thereby suppressing ferroptosis (22). Fourth, enhanced ubiquitination-mediated degradation of ferroptosis regulators occurs. E6 and E7 upregulate and promote acetylation of the TUBORF peptide, encoded by the *tuba3fp*, which accelerates ubiquitin-dependent degradation of critical ferroptosis inducers such as IRGQ, further inhibiting ferroptosis (**Figure 2**) (23). Through these anti-ferroptotic mechanisms, SCC cells evade immune surveillance and clearance, facilitating their survival and malignant progression.

#### 2.2.2 The role of the KRAS signaling pathway in cervical cancer cells’ resistance to ferroptosis

Kirsten rat sarcoma viral oncogene (KRAS) is a member of the RAS gene family, located on the short arm of human chromosome 12 (12p1.1-pter). The gene spans approximately 35 kb and encodes a small GTPase protein known as the KRAS protein. Under physiological conditions, the normal KRAS protein undergoes transiently activation to stimulate downstream signaling pathways—including MAPK and PI3K/AKT—that regulate essential cellular processes such as proliferation, differentiation, metabolism, and apoptosis. However, mutations in KRAS result in constitutive activation of the protein. This leads to uncontrolled cell proliferation, aberrant angiogenesis, and suppression of apoptosis, ultimately promoting tumorigenesis (24).

KRAS promotes ferroptosis resistance primarily through two interconnected mechanisms. First, mutant KRAS activates the Nrf2 antioxidant pathway. Constitutively active KRAS protein activates the downstream MAPK signaling pathway, which in turn promotes the activation of the transcription factor Nrf2. This leads to the upregulation of antioxidant-related gene expression (25). The action of these gene products effectively inhibits ferroptosis and reduces the sensitivity of tumor cells to ferroptosis. Second, KARS enhances SLC7A11-mediated glutathione synthesis. Mutant KRAS upregulates the expression of the cystine/glutamate antiporter SLC7A11, thereby enhancing cellular cystine uptake. This elevated cystine promotes the synthesis of GSH (26), an essential cofactor for GPX4. Elevated GSH levels enhance GPX4 activity, facilitating the clearance of lipid peroxides and suppressing the initiation of ferroptosis (**Figure 2**).

In summary, KRAS mutation and subsequent overexpression are key factors enabling cervical cancer cells to acquire ferroptosis resistance. This adaptive resistance allows cancer cells to survive under the high oxidative stress in the tumor microenvironment, evade clearance via ferroptosis, and ultimately support malignant progression (15).

#### 2.2.3 The translational value of ferroptosis-related genes in prognostic assessment and treatment strategy selection for HPV-associated cervical cancer

Recent clinical cohort studies have revealed that ferroptosis-related genes are dysregulated in HPV-associated cervical cancer and are closely associated with disease progression, the immune microenvironment, and patient prognosis.

SLC7A11, functioning as a cystine/glutamate antiporter, shows that its high expression is significantly correlated with shortened overall survival in cervical cancer patients. Further analysis indicated that tumors with high SLC7A11 expression exhibit increased infiltration of activated mast cells and M0 macrophages, alongside a decreased proportion of resting dendritic cells within the tumor microenvironment, suggesting it may promote tumor progression by remodeling the immune microenvironment. Furthermore, patients in this group demonstrated a higher tumor mutational burden (TMB) score, indicating potential increased sensitivity to immunotherapy (27, 28).

Regarding disease progression, TFRC expression is elevated in cervical cancer and positively correlates with T stage and N stage. Prognostically, patients with high TFRC expression had significantly shorter overall survival (OS), disease-specific survival (DSS), and progression-free interval (PFI). Mechanistically, high TFRC expression negatively correlates with the infiltration levels of key anti-tumor immune cells such as CD8^+^ T cells and positively correlates with immunosuppressive cells like Th2 cells, suggesting it promotes disease progression and poor prognosis by modulating the tumor immune microenvironment (29, 30).

STEAP3 is a metalloreductase and a crucial regulator of ferroptosis. It can inhibit p53 via the p53-SLC7A11 axis, thereby promoting SLC7A11 expression and conferring a ferroptosis-resistant effect on cancer cells (31). Clinical cohort studies show that patients with high STEAP3 expression have poorer OS and progression-free survival (PFS). Fundamental research further confirms that inhibiting STEAP3 enhances the sensitivity of cervical cancer cells to platinum-based chemotherapy drugs, suggesting its involvement in chemoresistance (32, 33).

Nrf2, a key antioxidant transcription factor, was found to be upregulated in cervical cancer patients with lymph node metastasis (34, 35), indicating its potential important role in the metastatic process of cervical cancer, possibly related to ferroptosis resistance.

Collectively, these clinical and mechanistic studies demonstrate that ferroptosis-related markers hold potential translational value for prognostic assessment and guiding treatment strategy selection in cervical cancer.

#### 2.2.4 Therapeutic strategies targeting ferroptosis for HPV-associated cervical cancer

Ferroptosis plays a critical role in the progression of HPV-associated cervical cancer, highlighting its considerable therapeutic potential for this malignancy.

First, targeting ferroptosis resistance in tumor cells represents a promising novel strategy for cervical cancer treatment. For instance, HPV-16 enhances the ferroptosis resistance by activating c-Myc gene expression. Conversely, in cervical squamous cell carcinoma (CSCC), HOXA5-mediated transcriptional downregulation of the ferroptosis suppressor SLC7A11 significantly increases ferroptosis sensitivity and promotes tumor cell death (36). Similarly, in triple-negative breast cancer (TNBC), high GPX4 expression mediates ferroptosis resistance, and the use of GPX4 inhibitors effectively induces ferroptosis and exerts significant antitumor effects (37).

Second, the combination of ferroptosis inducers with traditional antitumor drugs demonstrates superior efficacy compared to monotherapy. In TNBC, combination a GPX4 inhibitor and anti-PD-1 immunotherapy demonstrates better outcomes than either treatment alone (37). In KRAS-mutant colorectal cancer, the addition of ferroptosis inducers to cetuximab (an anti-EGFR monoclonal antibody) overcomes the limited efficacy of cetuximab-based chemotherapy imposed by KRAS mutation, effectively suppressing tumor growth and lymph node metastasis (38). Given that KRAS-mutant cervical cancer also shows reduced sensitivity to platinum/taxane-based chemotherapy, combining ferroptosis inducers with conventional chemotherapeutic agents offers a promising new approach to overcome this therapeutic resistance.

Finally, targeting ferroptosis-related pathways to induce ferroptosis in tumor cells has great potential for the treatment of cervical cancer. In KRAS-mutant colorectal cancer, inhibition of the AKT/mTOR signaling pathway enhances tumor cells sensitivity to ferroptosis and significantly inhibit tumor progression (39). Based on similar mechanisms, targeting key pathways such as Nrf2 and MAPK signaling may provide a breakthrough in the treatment of HPV-related cervical cancer.

In summary, strategies that targeting ferroptosis to overcome treatment resistance, synergize with traditional therapies, and modulate key pathways offer a promising new treatment paradigm for patients with HPV-related cervical cancer. Further exploration of the ferroptosis regulatory network in cervical cancer will promote the development of precision treatment strategies.

However, the molecular mechanism by which HPV regulates immune function and its interaction with ferroptosis pathway have not been fully elucidated. Future research needs to further explore the dynamic regulation process of HPV-host interaction. Building on this foundation, it will be essential to develop therapeutic strategies targeting ferroptosis pathways, clarify the pharmacological mechanisms of drug candidates, and investigate the underlying causes of drug resistance.

### 2.3 S. aureus

*S. aureus* is a Gram-positive coccus widely distributed in the natural environment. Colonization of this pathogen within the reproductive system can lead to a range of inflammatory conditions, including vulvitis and pelvic inflammatory disease. These severe clinical manifestations constitute a significant risk factor for infertility, underscoring the importance of effective management. Antibiotic therapy remains the primary treatment for *S. aureus* infections in the reproductive system. However, the organism’s considerable environmental adaptability, capacity for biofilm formation, and the extensive (sand often inappropriate) use of antibiotics have contributed to the emergence of multidrug-resistant strains, such as methicillin-resistant *S. aureus* (MRSA) and vancomycin-resistant *S. aureus* (VRSA). As a result, antimicrobial resistance in *S. aureus* has become a critical challenge in clinical management. Notably, recent studies have revealed that modulation of ferroptosis-related signaling pathways can confer therapeutic effects in models of *S. aureus*-induced endometritis and mastitis (28, 30). This finding provides a valuable conceptual framework for developing novel therapeutic strategies targeting *S. aureus* infections of the reproductive system.

#### 2.3.1 Ferroptosis in *S. aureus*-induced endometritis

Endometritis is an inflammatory disease of the endometrium caused by pathogenic infection or mechanical injury. It constitutes a component of the pelvic inflammatory disease spectrum. It predominantly affects sexually active women of reproductive age, with lower incidence observed in postmenopausal or premenarchal individuals. Left untreated, endometritis can result in serious complications, including infertility and ectopic pregnancy, among others. *S. aureus* is frequently identified as an etiological agent in the pathogenesis of this condition. In *S. aureus*-induced endometritis, endometrial cells exhibit pronounced ferroptotic effects, marked by significant oxidative stress imbalance-evidenced by GSH depletion, accumulation of lipid peroxides, and iron overload- as well as mitochondrial morphological alterations including reduced cristae and increased membrane density (28). The ferroptosis is primarily mediated by four interconnected mechanisms.

**Figure 3:**
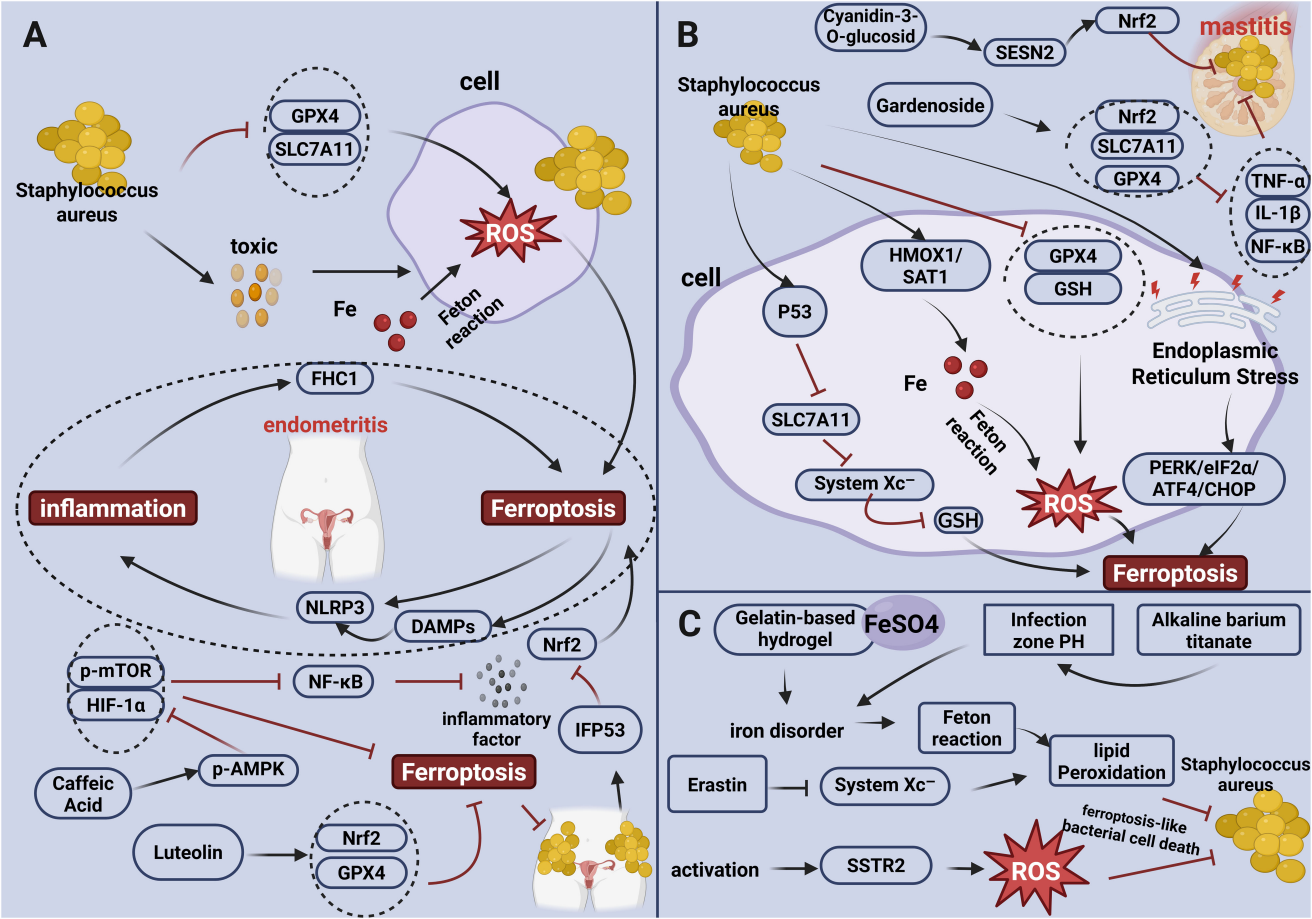
Schematic diagram of the correlation between ferroptosis and *S. Aureus* infection. **(A)** Ferroptosis Mechanisms and Intervention in Endometritis. In endometritis induced by *S. aureus* infection, the pathogen suppresses the expression of GPX4 and SLC7A11 and secretes toxins that disrupt cell membranes, promoting iron influx and generating ROS via the Fenton reaction, thereby triggering ferroptosis. Ferroptosis further exacerbates the inflammatory response by activating the NLRP3 inflammasome and releasing DAMPs. In turn, the inflammatory response upregulates FTH1 expression, forming a vicious cycle of “inflammation–ferroptosis” that aggravates endometrial damage. Caffeic Acid alleviates ferroptosis and inflammation by activating p-AMPK and inhibiting HIF-1α, p-mTOR, and NF-κB-mediated cytokine release. Luteolin, on the other hand, mitigates ferroptosis by upregulating Nrf2 and GPX4 expression. **(B)** Ferroptosis Pathways and Pharmacological Targeting in Mastitis. Following *S. aureus* infection in mammary cells, the pathogen promotes ferroptosis by upregulating HMOX1/SAT1 to increase iron uptake and ROS generation, inhibiting the p53-mediated SLC7A11/system Xc^−^/GSH pathway, and downregulating GPX4 and GSH. These changes induce endoplasmic reticulum stress and activate the PERK/eIF2α/ATF4/CHOP signaling axis. Cyanidin-3-O-glucoside alleviates mastitis through the SESN2/Nrf2 pathway, while Gardenoside exerts anti-inflammatory and anti-ferroptotic effects by activating the Nrf2/SLC7A11/GPX4 signaling pathway and inhibiting TNF-α, IL-1β, and NF-κB. **(C)** Antibacterial Strategy Targeting Bacterial Ferroptosis Like Bacterial Cell Death. A gelatin-based hydrogel loaded with ferrous sulfate disrupts intracellular iron homeostasis in *S. aureus*, induces lipid peroxidation, and leads to ferroptosis-like bacterial cell death. Erastin triggers a ferroptosis-like bacterial cell death by inhibiting the bacterial system Xc^−^, resulting in accumulation of lipid peroxides. Activation of the SSTR2 receptor in *S. aureus* promotes ROS generation and induces ferroptosis-like death. Alkaline barium titanate can modulate the local pH, disrupt iron metabolism, enhance lipid peroxidation, and thereby induce ferroptosis-like bacterial cell death.

First, *S. aureus* infection significantly downregulates the expression of GPX4 and SLC7A11, two key proteins that inhibit lipid peroxidation and ferroptosis (30). Second, toxins secreted by *S. aureus* damage endometrial cell membranes, enhancing iron influx and fostering Fenton reactions that accelerate lipid peroxide accumulation (28). Third, *S. aureus* infection upregulates Interferon-Induced Protein 35 (IFP35), which promotes ferroptosis by facilitating the ubiquitin-mediated degradation of Nrf2, a suppressor of ferroptosis (31). Fourth, a vicious cycle ensues wherein ferroptosis and inflammation mutually reinforce each other. Inflammatory cytokines stimulate the expression of ferroptosis-related proteins such as Ferritin Heavy Chain 1, thereby inducing ferroptosis (32). In turn, ferroptosis damage activates the NLRP3 inflammasome, establishing a positive feedback loop that amplifies inflammatory signaling (33). Furthermore, damage-associated molecular patterns (DAMPs) released during ferroptosis activate the NLRP3 inflammasome, exacerbating the inflammatory cascade (**Figure 3A**) (34).

Targeting ferroptosis-related genes has emerged as a promising therapeutic strategy to mitigate *S. aureus*-induced endometritis. By inhibiting ferroptosis in uterine endothelial cells, this approach disrupts the detrimental crosstalk between ferroptosis and inflammation. Current research demonstrates that Luteolin inhibits ferroptosis and alleviates *S. aureus*-caused endometrial inflammation by activating the Nrf2/GPX4 signaling pathway, thereby enhancing antioxidant capacity and limiting infectious pathology (28). Similarly, caffeic acid effectively alleviates endometritis by upregulating p-AMPK expression while inhibiting HIF-1α and p-mTOR expression, leading to inhibition of both NF-κB-mediated inflammatory cytokine release and ferroptosis (35). Additionally, siRNA-mediated knockdown of PTGS2 has been shown to block inflammatory cytokine release and IL-17A-induced ferroptosis, concurrently mitigating both processes (**Figure 3A**) (36).

#### 2.3.2 Ferroptosis and *S. aureus*-induced mastitis

Mastitis is an inflammatory condition affecting the mammary gland tissue. Exotoxins secreted by *S. aureus* play a critical role in its pathogenesis. Certain exotoxins, such as toxic shock syndrome toxin-1 (TSST-1) and staphylococcal enterotoxins (SEs), act as superantigens that activate large numbers of T cells, resulting in excessive release of pro-inflammatory cytokines including TNF-α and IL-6, which cause severe inflammatory damage in mammary tissue. Leukocidin lys immune cells, thereby weakening host defense mechanisms and promoting chronicity of mastitis. Additionally, α-Hemolysin disrupts mammary epithelial cell membranes, facilitating bacterial invasion (40).

The inflammatory response is the key mechanism in the development and progression of mastitis. *S. aureus* infection activates the NF-κB signaling pathway, leading to the release of inflammatory cytokines. These cytokines further downregulate the expression of tight junction proteins such as ZO-1, Occludin, Claudin-3in mammary tissue, thereby compromising the blood-milk barrier. This impairment results in two major consequences: serum protein leakage into the milk and increased pathogen invasion into mammary tissue (41).

*S. aureus*-induced mastitis is accompanied by significant ferroptosis in mammary cells. The mechanisms involve several pathways. First, *S. aureus* infection markedly upregulates the expression of HMOX1 and SAT1. Excessive HMOX1 expression increases intracellular free iron, promoting ROS generation via the Fenton reaction, while SAT1 augments hydrogen peroxide production, collectively inducing oxidative stress and ferroptosis (42). Second, *S. aureus* infection depletes GSH and inhibits GPX4 activity within mammary tissue cells, impairing the cellular antioxidant defense system. The resulting redox imbalance allows accumulation of ROS, leading to oxidative damage to organelles and membranes and ultimately triggering ferroptosis (42, 43). Third, *S. aureus* infection triggers endoplasmic reticulum stress in mammary tissue cells, activating the PERK/eIF2α/ATF4/CHOP signaling axis. This activation promotes ferroptosis, as evidenced by mitochondrial shrinkage and dysregulation of key ferroptosis genes, such as increased ACSL4 expression and decreased GPX4 expression, causing damage to the mammary tissue (44). Finally, *S. aureus* infection significantly reduces SLC7A11 protein expression and stabilizes p53 activity. Activated p53 binds to the SLC7A11 promoter, repressing its transcription. Downregulation of SLC7A11 impairs system Xc^−^ function, leading to reduced antioxidant capacity and induction of ferroptosis (**Figure 3B**) (45).

Targeting ferroptosis represents a novel therapeutic strategy for treating *S. aureus*-induced mastitis. Research indicates that Cyanidin-3-O-glucoside activates SESN2, a stress-inducible protein that regulates oxidative stress and metabolism. SESN2 activation upregulates Nrf2 expression, enhances antioxidant responses and suppresses both the inflammatory response and ferroptosis, thereby effectively alleviating mastitis (46). Similarly, Gardenoside alleviates *S. aureus*-induced mastitis by activating the Nrf2/SLC7A11/GPX4 signaling pathway, which reduces pro-inflammatory cytokines (TNF-α, IL-1β) expression and neutrophil infiltration. It also inhibits NF-κB phosphorylation and ferroptosis by diminishing lipid peroxidation and enhancing antioxidant activity (**Figure 3B**) (47).

#### 2.3.3 Inducing a ferroptosis-like bacterial cell death in *S. aureus* represents a novel strategy to overcome its antibiotic resistance

Recent studies indicate that *S. aureus* can be induced to undergo a form of cell death resembling ferroptosis, herein referred to as a ferroptosis-like bacterial cell death. This process is iron-dependent, involved uncontrolled lipid peroxidation, and the failure of key antioxidant systems, ultimately leading to disruption of cell membrane integrity. Although *S. aureus* lacks both the canonical GPX4 protein and mitochondria, the observed cell death phenotypically mirrors eukaryotic ferroptosis (48). Targeting this ferroptosis-like bacterial cell pathway represents a promising therapeutic strategy to overcome *S. aureus* antibiotic resistance.

The induction of this effect in *S. aureus* can be achieved through several interconnected mechanisms. Primarily, iron homeostasis in *S. aureus* is disrupted. Studies demonstrate that a gelatin-based hydrogel loaded with ferrous sulfate (FeSO_4_) promotes intracellular Fe 2^+^ accumulation in antibiotic-resistant *S. aureus*, inducing iron dyshomeostasis and activating the Fenton reaction. This results in a burst of ROS and lipid peroxidation, ultimately inducing the ferroptosis-like bacterial cell death (49). Secondly, the antioxidant defense system in *S. aureus* is impaired. For instance, employing zeolitic imidazolate framework-8 (ZIF-8) to deliver Erastin inhibits system Xc^−^ function, depleting intracellular GSH and rendering the bacteria incapable of clearing lipid peroxides, thereby inducing the ferroptosis-like bacterial cell death (50). Additionally, activation of *S. aureus* SSTR2 receptor in induces metabolic dysregulation, leading to the massive production of ROS that damages cell membrane phospholipids (specifically polyunsaturated fatty acids, PUFAs). Lastly, modulation of the local pH environment using alkaline barium titanate microzone that disrupts intrabacterial iron metabolism, promotes lipid peroxidation, and damages bacterial mesosomes—functional analogues of mitochondria—further inducing the ferroptosis-like bacterial cell death (**Figure 3C**) (51).

*S. aureus* resists host-induced ferroptosis-like cell death through its intrinsic antioxidant systems and iron metabolism regulatory network. Understanding these defense mechanisms provides the theoretical foundation for developing novel antibacterial drugs. The peroxidase KatA is a core component of the *S. aureus* antioxidant defense system. It is constitutively expressed under aerobic conditions, assisting the bacterium in clearing H_2_O_2_, and is also an essential factor for *S. aureus* to acquire pre-adaptive resistance under microaerophilic conditions (52). KatA also contributes to regulating *S. aureus* resistance to quinolone and β-lactam antibiotics. These antibiotics induce significant accumulation of ROS within the bacteria, and KatA effectively blocks the ROS chain reaction by decomposing ROS, thereby mitigating oxidative damage and enhancing survival rates (53). Studies have shown that flavonoid extracts can exert bactericidal effects by inhibiting KatA protein activity within MRSA, compromising its antioxidant stress capacity (54). This suggests that targeting KatA to induce ferroptosis-like death in *S. aureus* could be a viable strategy against MRSA. The iron uptake regulator (Fur) is a key transcription factor controlling intracellular iron homeostasis and oxidative stress. When Fe²^+^ levels are sufficient, Fur binds Fe²^+^ and then binds to the Fur box in the promoter regions of target genes, repressing the transcription of a series of genes, including the iron uptake system, thereby limiting free iron accumulation and preventing the Fenton reaction (55). The labile iron pool (LIP) content is significantly increased in Fur loss-of-function mutants (*Δfur*), indicating that Fur deletion leads to constitutive activation of iron uptake, ultimately resulting in intracellular iron overload. This underscores Fur’s central role in preventing iron toxicity (56). Under iron-deficient conditions, Fur activates the expression of high-affinity iron transporters and initiates an iron-sparing stress response via the sRNA IsrR, which includes repression of katA expression, thereby moderately weakening the antioxidant stress capacity to maintain iron metabolic balance (57). Therefore, targeting the Fur regulatory network can disrupt bacterial iron homeostasis and enhance its susceptibility to ferroptosis-like death. Furthermore, studies have found that *S. aureus* mutant strains with high c-di-AMP expression, while exhibiting thickened cell walls and enhanced resistance to β-lactam antibiotics (such as oxacillin), show a significantly reduced capacity to withstand oxidative stress (58). This phenomenon suggests that such resistant strains might be more vulnerable to oxidative stress-mediated killing, offering new avenues for treating refractory infections caused by them.

Notably, induction of the ferroptosis-like bacterial cell death demonstrates potent bactericidal activity not only against Gram-positive bacteria such as *S. aureus*, but also Gram-negative species including *Pseudomonas aeruginosa* (59). This broad-spectrum efficacy highlights its potential as a novel strategy to address the escalating challenge of bacterial antibiotic resistance, particularly multidrug-resistant infections. However, as prokaryotes, bacteria fundamentally differ from eukaryotic cells in their cellular structure and metabolic pathways. Future research should therefore prioritize elucidating the unique iron homeostasis regulatory mechanisms and antioxidant defense networks in prokaryotic systems. A deeper understanding of these pathway is essential to clarify the molecular basis of the ferroptosis-like bacterial cell death in bacteria and to facilitate its translation into targeted therapeutic applications.

### 2.4 SARS-CoV-2

SARS-CoV-2 is an enveloped, positive-sense single-stranded RNA virus belonging to the Betacoronavirus genus (60). Since its emergence in 2020, the COVID-19 pandemic ihas continued to pose a severe threat to global public health, with ongoing evolving variants persist in causing significant harm to human health. The virus utilizes angiotensin-converting enzyme 2 (ACE2) as its primary receptor, which is widely distributed in multiple organs including the kidneys, testes, intestines, lungs, retina, placenta, cardiovascular system, adipose tissue, and central nervous system (61, 62), demonstrating the virus’s multi-organ targeting capability.

A key aspect of SARS-CoV-2 pathogenesis is its strong induction of oxidative stress. The virus directly damages host cellular structural proteins and membrane lipid structures, and triggers a cytokine storm leading to immune dysregulation and hyperinflammatory responses, collectively contributing to a range of clinical manifestations (63). Notably, oxidative stress and inflammatory responses disrupt intracellular iron metabolism homeostasis, thereby driving phospholipid peroxidation. Excessive lipid peroxidation induces membrane damage and activates ferroptosis (64). The reproductive system (e.g., testes, ovaries) is highly sensitive to oxidative stress and iron metabolism dysregulation. Accumulating evidence indicates that SARS-CoV-2 infection of the reproductive system is associated with various pathologies, including male infertility (65), hypotestosteronemia, orchitis and epididymitis, erectile dysfunction, placentitis (66), preeclampsia, preterm birth, and stillbirth (67). Therefore, elucidating the mechanisms underlying the interaction between SARS-CoV-2 infection and ferroptosis in the reproductive system represents an important avenue for further research.

The diverse pathologies arising from SARS-CoV-2 infection of the reproductive system, while clinically heterogeneous, share a common regulatory axis in their underlying ferroptosis mechanisms, alongside tissue-specific variations. The IL-6/NF-κB signaling pathway-mediated inflammatory-ferroptosis cycle constitutes a core common pathway triggered by the virus. The cytokine storm (particularly IL-6) unleashed following viral entry activates NF-κB, which not only exacerbates local inflammation but also systemically disrupts iron metabolic homeostasis. This promotes hepcidin expression, leading to functional iron deficiency and tissue iron accumulation, thereby creating a prerequisite for ferroptosis initiation. On this common foundation, the intrinsic properties of different reproductive tissues determine their susceptibility to ferroptosis and the divergence in specific mechanisms. For instance, testicular tissue highly expresses long-chain polyunsaturated fatty acids sensitive to oxidative stress, and its blood-testis barrier creates a unique immune microenvironment. The placenta undergoes a distinctive hypoxia-reperfusion process, and its trophoblast cells are metabolically highly active. Ovarian granulosa cells, conversely, are sensitive to hormonal fluctuations and metabolic changes. These differences explain why the same inflammatory trigger ultimately acts upon distinct, disease-specific key targets—such as Plin4 in male infertility, the MLL1/RBM15 epigenetic axis in pre-eclampsia, and FZD7 in ovarian cancer—leading to divergent pathological outcomes.

Based on this framework, the following sections will detail the specific mechanisms of ferroptosis in each respective disease. The association between ferroptosis and SARS-CoV-2 infection-induced reproductive system infection is shown in (**Table 1**).

**Table 1:** Association of ferroptosis with SARS-CoV-2 infection-induced reproductive disorders.

Disease	Ferroptosis-related features		Main molecular mechanisms	Potential therapeutic strategies
Male Infertility	Decreased GPX4, SLC7A11, Increased ROS, MDA, Fe²^+^		Membrane lipid disruption, Inflammation-iron dysregulation cascade, Impaired NO synthase complex function promoting ROS release	Ferroptosis inhibitors (Fer-1, DFO); Natural compounds (e.g., Ursolic acid, Platycodin, Cordycepin, Myristic acid, Sinomenium acutum, Mango, Cuscuta, Lycium); Nrf2 pathway activation
Testosterone Deficiency	Plin4 upregulation, Mitochondrial dysfunction, Impaired cholesterol metabolism		Plin4 accumulation driving ferroptosis & mitochondrial damage, CYP11A1 inhibition impairing testosterone synthesis	Ferroptosis inhibitors (Fer-1, DFO), Plin4-specific siRNA/shRNA inhibitors, Mitochondrial function repair agents
Orchitis/ Epididymitis	Inflammatory cell infiltration, Elevated ROS, TNF-α, Blood-testis barrier disruption		Macrophage polarization releasing ROS/TNF-α; Inhibition of glutathione synthase, Inflammation-ferroptosis positive feedback loop, Blood-testis barrier damage	Anti-inflammatory drugs (TNF-α inhibitors), Ferroptosis inhibitors, Antioxidants (e.g., GSH precursors), Blood-testis barrier protection
Erectile Dysfunction	Ferroptosis in endothelial & smooth muscle cells, Decreased NO synthesis, TGF-β/Smad activation		Endothelial ferroptosis causing vasodilatory dysfunction, Smooth muscle ferroptosis leading to collagen deposition, structural remodeling, and fibrosis	Ferroptosis inhibitors, Hemodynamic improvement, TGF-β/Smad pathway antagonists
Preeclampsia	Increased serum iron, ferritin, MDA, Decreased placental GPX4, GSH, Abnormal mitochondria		MLL1/RBM15/TRIM72/ADAM9 axis, miR-30b-5p/SLC7A11-PAX3 axis, High Lpcat3, Sat1 expression	MLL1/RBM15/TRIM72/ADAM9 axis inhibitors, miR-30b-5p antagonist, PAX3 or SLC7A11 agonists, Ferroptosis inhibitors (Ferrostatin-1)
Polycystic Ovary Syndrome	Increased serum ferritin, Elevated ovarian MDA, decreased GPX4, SLC7A11/ACSL4 dysregulation		Iron dysregulation (TFRC↑, FPN1↓, NCOA4↑), Impaired antioxidant system, Dysregulated Hippo-YAP, NF-κB, AMPK/mTOR pathways, Hyperandrogenism-ferroptosis cycle	Iron chelators (DFO), Lipid peroxidation inhibitors (Ferrostatin-1, Liproxstatin-1); GPX4 activators (Selenium), Lifestyle intervention; Metformin (AMPK activator), Rosiglitazone (PPARγ agonist)
Ovarian Cancer	Iron dysregulation, aberrant GPX4 expression, Enhanced lipid peroxidation		Nrf2/HERC2/VAMP8/NCOA4 axis, FZD7/β-catenin/Tp63/GPX4 axis, High SCD1 expression	Ferroptosis inducers, PARP inhibitor combination therapy, Immunotherapy combination, Targeting FZD7, Nrf2 pathways
Spontaneous Abortion	Upregulated HMGB1, ACSL4, Increased MDA, Decreased GSH in placental tissue		HMGB1/ACSL4 axis activation, Hypoxia/HIF1α-SUMO/NCOA4 axis, IGF2BP3-mediated TFR1 mRNA stabilization	HMGB1 inhibitors, lnc-HZ06 antagonist, Antioxidants (e.g., Vitamin E), Ferroptosis inhibitors
Endometriosis	Elevated iron, ROS in peritoneal/follicular fluid, Decreased GPX4, Increased TFRC, Ferritinophagy	ACE2 downregulation leading to angiotensin II accumulation & oxidative stress, Viral infection-induced cytokine storm exacerbating inflammation via inflammation-ferroptosis feedback, Inflammatory cytokines suppressing GPX4 & promoting iron uptake		Iron chelators (Deferoxamine), Antioxidants (Vitamin E); Anti-inflammatory therapy (e.g., IL-6 inhibitors), Targeting SLC7A11/GPX4

#### 2.4.1 Male infertility

Male infertility is clinically defined as the failure to achieve pregnancy after one year of regular unprotected intercourse in couples of reproductive age (68), Its pathological basis often involves impaired spermatogenic secondary to primary testicular failure. The testicular microenvironment plays a crucial regulatory role in supporting spermatogenesis, and dysfunction of any cellular component within this microenvironment can contribute to male infertility.

Recent studies indicate that ferroptosis significantly contributes to SARS-CoV-2-associated male infertility through multiple mechanisms. It is important to note that the causal role of ferroptosis is supported by interventional studies: in mouse models, the ferroptosis inhibitor Ferrostatin-1 (Fer-1) was shown to alleviate testicular damage and improve sperm quality, confirming its status as a key pathogenic mechanism (69). First, SARS-CoV-2 infection disrupts membrane lipid disruption and promotes ferroptosis. The virus directly damages testicular cell membrane lipid structures, leading to intracellular iron accumulation and upregulation of acyl-CoA synthetase long-chain family member 4 (ACSL4), which facilitates the integration of polyunsaturated fatty acids (PUFAs) into cell membranes, thereby enhancing their susceptibility to lipid peroxidation. Depletion of GPX4 in Sertoli cells disrupts the blood-testis barrier, resulting in cell death and ultimately spermatogenic arrest (70, 71). Second, SARS-CoV-2 triggers an inflammation-iron dysregulation cascade. This essentially represents the manifestation of the common IL-6/NF-κB pathway within testicular tissue. Viral invasion upregulates pro-inflammatory cytokine such as IL-6, which disrupts iron homeostasis and causes intracellular iron overload. This excess iron overload promotes ROS generation via the Fenton reaction. ROS subsequently attack PUFAs-rich sperm cell membranes, inducing lipid peroxidation and impairing male reproductive function (72, 73). These factors collectively constitute the basis for the high susceptibility of the testes to ferroptosis.

Ferroptosis has also been implicated in other common causes of male infertility, including asthenospermia and varicocele. Semen analysis from asthenozoospermic patients reveals significantly reduced expression of GPX4 and SLC7A11, along with elevated ferroptosis markers such as ROS, malondialdehyde (MDA) and intracellular iron levels. expression and sperm motility, indicating that GPX4 downregulation is a key mechanism in ferroptosis-related male infertility (74). Varicocele (VCL), characterized by abnormal dilation of the Pompini-like venous plexus, impairs testicular thermoregulation and induces chronic hypoxic stress, leading to defective spermatogenesis (74). Heat and hypoxic stress directly activate mitochondrial electron transport chain complex III, promoting ROS release. At the same time, NO is released in testicular cells and endothelial cells, which inhibits the function of complexes I and IV through nitrosylation modification, and further releases ROS, which synergistically induces ferroptosis (75).

Targeting ferroptosis represents a promising therapeutic strategy for male infertility. Ferroptosis inhibitors such as Ferrostatin-1 (Fer-1) and deferoxamine (DFO) have been shown to mitigate testicular oxidative stress and lipid peroxidation. In oligospermia models, Fer-1 improves sperm quality by upregulating GPX4 and activating the Nrf2 signaling pathway. Additionally, several natural compounds exhibit therapeutic value through ferroptosis modulation and antioxidant enhancement, including ursolic acid, platycodin D, cordycepin, myristic acid, limonium sinense extracts, mango polyphenols, cuscuta flavonoids, and lycium barbarum polysaccharides (74).

#### 2.4.2 Testosterone deficiency syndrome (male hypogonadism)

Testosterone Deficiency Syndrome (male hypogonadism) is defined as a clinical syndrome characterized by persistently reduced serum testosterone levels accompanied by characteristic symptoms (76). Emerging evidence indicates that beyond direct damage to testicular Leydig cells, ferroptosis significantly contributes to SARS-CoV-2-associated testosterone deficiency. This is demonstrated by the restoration of testosterone synthesis achieved through targeting the ferroptosis pathway, particularly by inhibiting Plin4.

SARS-CoV-2 infection upregulates perilipin 4 (Plin4) expression in Leydig cells, leading to dysregulated lipid droplet metabolism. ExcessivePlin4 accumulation drives ferroptosis initiation, leading to structural and functional mitochondrial impairment (71). The damaged mitochondria are unable to adequately support cholesterol metabolism, resulting in the inhibition of cholesterol to conversion pregnenolone, a process mediated by the mitochondrial inner membrane enzyme CYP11A1. Consequently, the subsequent conversion of pregnenolone to testosterone, which depends on enzyme systems located in the mitochondria and smooth endoplasmic reticulum, is severely attenuated. These metabolic disruptions collectively contribute to testosterone deficiency pathogenesis.

Targeting ferroptosis presents considerable therapeutic potential for testosterone deficiency. On one hand, ferroptosis inhibitors Fer-1 and DFO have been shown to effectively inhibit Plin4-mediated ferroptosis pathways, thereby restoring mitochondrial cholesterol metabolic function. On the other hand, the development of novel gene therapeutic approaches is particularly critical. Plin4-targeted siRNA and shRNA delivery systems can selectively suppress ferroptosis activation. Targeting the ferroptosis pathway—especially the Plin4-mitochondrial axis—not only reverses SARS-CoV-2-associated hypotestosteronemia but also represents a novel treatment framework for male hypogonadism more broadly.

#### 2.4.3 Testicular epididymitis

Testicular epididymitis is highly prevalent among adolescents, with viral pathogens being the most common causative agents (77). SARS-CoV-2 infection has been shown to disrupt the epididymal microenvironment and impair sperm maturation (65). The histological characteristics of testicular epididymitis include testicular interstitial hemorrhage, inflammatory cell infiltration around the seminiferous tubules, and vacuoleic degeneration of epididymal epithelial cells.

Emerging evidence indicates that ferroptosis plays an important role in the pathogenesis of testicular epididymitis. SARS-CoV-2 infection induces polarization of testicular macrophages, triggering an explosive release of ROS and pro-inflammatory factor TNF-α, etc., ROS promotes lipid peroxidation via Fenton reaction, while TNF-α inhibits the expression of glutathione synthetase. These processes act synergistically to induce ferroptosis in spermatogenic epithelial cells.

Ferroptosis cells release DAMPs, which further activates NLRP3 inflammasome and exacerbate the systemic inflammatory response. This leads to loss of the blood-testis barrier integrity and promotes the infiltration of peripheral immune cells. Together, these events establish a self-amplifying cycle of “inflammation-ferroptosis,” ultimately driving the initiation and progression of testicular epididymitis.

#### 2.4.4 Erectile dysfunction

Erectile dysfunction (ED) is significantly increased in patients with long COVID. Ultrasound examinations reveal characteristic hemodynamic abnormalities within the penile corpus cavernosum, and the underlying molecular mechanism involves dual-tissue injury mediated by ferroptosis.

First, endothelial ferroptosis leads to impaired vasodilation. Ferroptosis in cavernous endothelial cells leads to reduced synthesis of NO and disruption of the cGMP signaling pathway, ultimately compromising vasodilatory function.

Second, ferroptosis in corpus cavernosum smooth muscle cells contributes to corpus cavernosum structural remodeling. Lipid peroxide accumulation triggered by smooth muscle ferroptosis activates the TGF-β/Smad signaling pathway. This induces a fibrotic phenotype transition of smooth muscle cells, increases collagen deposition, reduces tissue compliance, and impairs venous occlusion function. Together, these two mechanisms synergistically lead to the development of organic erectile dysfunction.

#### 2.4.5 Preeclampsia

PE is a pregnancy complication with a significantly elevated risk following SARS-CoV-2 infection. Its key pathological features involve impaired extravillous cytotrophoblasts (EVCTs) function and incomplete remodeling of uterine spiral arteries. The disease originates from placental abnormalities: Between 8–10 weeks post-fertilization, the placenta undergoes a critical physiological hypoxia-reperfusion transition. This process is initially marked by complete obstruction of the spiral arteries, resulting in a low-oxygen, low-glucose environment. Subsequent recanalization of these vessels permits a massive influx of maternal blood, leading to abrupt elevations in oxygen levels and iron concentration. In response, the placenta secret increased levels of anti-angiogenic factors, notably soluble fms-like tyrosine kinase-1 (sFlt-1) and soluble endoglin (sEng). These factors contribute to EVCT endothelial dysfunction, vasoconstriction of spiral artery, and immune dysregulation. Togrther, these events precipitate placental ischemia and systemic hypertension, ultimately exerting detrimental effects on both the mother and fetus (78, 79). The systemic inflammatory response triggered by SARS-CoV-2 (via the IL-6/NF-κB pathway) amplifies the inherent oxidative stress associated with the physiological “hypoxia-reperfusion” transition in the placenta. Furthermore, placental trophoblast cells are particularly sensitive to disruptions in iron metabolism due to their high iron demand and unique metabolic characteristics during placental development. This heightened sensitivity renders the placenta a vulnerable organ for ferroptosis under SARS-CoV-2 infection. Additionally, studies suggest that ferroptosis may contribute to the pathogenesis of pre-eclampsia, and interventions targeting the proposed pathways provide compelling causal evidence linking the disease to ferroptosis.

Current research has elucidated multiple molecular mechanisms through which ferroptosis contributes to PE, with the MLL1/RBM15/TRIM72/ADAM9 axis and the miR-30b-5p/SLC7A11-PAX3 axis identified as key signaling pathways. Mixed Lineage Leukemia 1 (MLL1), a histone methyltransferase, is significantly upregulated in the placental villous tissues of PE patients. Inhibiting MLL1 in PE mouse models effectively ameliorates core PE symptoms, including hypertension and proteinuria, while concurrently attenuating ferroptosis-associated markers (e.g., reduced ROS, MDA, and Fe²^+^ levels; elevated GSH levels). Mechanistic studies reveal that MLL1 enhances H3K4me3 histone modification to promote expression of RBM15, an m6A methyltransferase that increases m6A modification on TRIM72 mRNA. This modification facilitates binding by YTH N6-methyladenosine RNA Binding Protein 2 (YTHDF2), accelerating TRIM72 mRNA degradation and reducing TRIM72 protein levels. TRIM72, an E3 ubiquitin ligase, normally targets ADAM9 (A Disintegrin and Metalloproteinase 9) for degradation. Its downregulation stabilizes ADAM9, promoting ferroptosis in trophoblasts and exacerbating PE symptoms (80).

In the miR-30b-5p/SLC7A11-PAX3 axis, miR-30b-5p directly targets the 3’ untranslated regions (3’UTRs) of both SLC7A11 (Solute Carrier Family 7 Member 11) and PAX3 (Paired Box 3), inhibiting their expression. Suppression of SLC7A11 reduces cystine uptake, thereby limiting GSH synthesis. Downregulation of PAX3 reduces the expression of its target gene ferroportin 1 (FPN1), impairing cellular iron export and resulting in the accumulation of intracellular free Fe²^+^. Together, these two pathways promote trophoblasts ferroptosis and contribute significantly to PE pathogenesis (81).

Based on these mechanisms, MLL1, RBM15, TRIM72, and ADAM9 represent potential therapeutic targets for PE. Consequently, miR-30b-5p inhibitors, ferroptosis inhibitors (such as Ferrostatin-1), and agonists of PAX3 or SLC7A11 show significant therapeutic potential. Future research should aim to identify additional downstream target of MLL1 and RBM15, develop specific inhibitors targeting the MLL1/RBM15/TRIM72/ADAM9 axis, optimize miR-30b-5p inhibitors for improved specificity and conduct preclinical safety evaluations of miR-30b-5p inhibitors.

#### 2.4.6 Polycystic ovary syndrome

PCOS is a complex endocrine and metabolic disorder affecting 6 - 20% of reproductive-aged women globally and represents a leading cause of anovulatory infertility. Patients often exhibit metabolic abnormalities including insulin resistance, obesity, and dyslipidemia, alongside psychological comorbidities such as depression and anxiety (82). The ovary, as the central organ for sex hormone regulation, relies heavily on stable energy metabolism and redox balance for the normal function of its granulosa cells. SARS-CoV-2 infection may disrupt this equilibrium by exacerbating systemic inflammation and oxidative stress. We therefore postulate that in PCOS patients with pre-existing insulin resistance and low-grade inflammation, the superimposed effect of the viral infection may more readily trigger ferroptosis in ovarian granulosa cells. It is important to note that while SARS-CoV-2 infection may exacerbate the pathophysiology of PCOS, the evidence directly linking SARS-CoV-2 to PCOS via ferroptosis is primarily correlative in nature, extrapolated from the established role of ferroptosis in PCOS itself and the virus’s capacity to amplify oxidative stress.

Clinical studies have demonstrated aberrant ferroptosis and oxidative stress markers in PCOS patients. Serum ferritin levels are significantly elevated and positively correlate with disease severity. Ovarian granulosa cells show increased MDA levels and reduced GPX4 activity. Key ferroptosis regulators, including SLC7A11 and ACSL4, are dysregulated in the granulosa cells of PCOS patients. These findings are supported by animal models. Di-(2-ethylhexyl) adipate (DEHA)-induced PCOS mouse exhibit elevated ovarian tissue iron content and characteristic morphological features of ferroptosis, such as mitochondrial shrinkage and increased membrane density (83, 84). Together, these findings indicate that ferroptosis is involved in PCOS progression.

Dysregulated iron metabolism plays a central role in promoting ferroptosis in PCOS. This includes upregulation of the transferrin receptor (TFRC), enhancing cellular iron uptake, downregulation of ferroportin (FPN1), inhibiting iron efflux and enhanced NCOA4-mediated ferritinophagy, leading to increased release of stored iron. These changes collectively elevate the intracellular labile iron pool (83, 84). Excess Fe²^+^ generates abundant ROS via the Fenton reaction, initiating lipid peroxidation and cellular damage (85). Furthermore, impaired antioxidant defenses promote ferroptosis. Downregulated SLC7A11 expression limited cystine uptake and GSH synthesis, compromising GPX4 activity. Additionally, reduced Nrf2 activity diminishes the transcriptional activation of antioxidant genes, weakening the cellular capacity to counteract ROS and lipid peroxides (83, 84, 86). The synergy between iron overload and antioxidant failure drives ferroptosis in granulosa cells. Multiple signaling pathways regulate ferroptosis in PCOS. The Hippo-YAP pathway is activated by n-3 polyunsaturated fatty acids (PUFAs), inhibiting YAP1 nuclear translocation and attenuating YAP1–Nrf2 interaction, thereby reducing antioxidant gene expression and increasing ferroptosis susceptibility (87). The NF-κB pathway is suppressed by miR-93-5p, leading to downregulation of SLC7A11 and GPX4, which promotes lipid peroxidation and cell death (86). The AMPK/mTOR pathway is modulated by metformin, which activates AMPK and inhibits mTOR, enhancing the SIRT3-mediated antioxidant response and alleviating ferroptosis (86).

A self-amplifying “hyperandrogenism-ferroptosis cycle” further exacerbates PCOS pathology. Dihydrotestosterone (DHT) upregulates NCOA4, enhancing ferritinophagy and increasing labile iron levels, thereby promoting ferroptosis. Ferroptotic granulosa cells release DAMPs that stimulate androgen production, forming a positive feedback loop. Hyperandrogenism and ferroptosis synergistically also synergize to worsen insulin resistance, amplifying metabolic dysfunction in PCOS (88, 89).

Targeted ferroptosis shows great potential in the treatment of PCOS. Firstly, ferroptosis inhibitors such as iron chelators (e.g., deferoxamine, DFO) c can reduce labile iron and improve granulosa cell function. Lipid peroxidation inhibitors such as antioxidants Ferrostatin-1 and Liproxstatin-1 can effectively inhibit lipid peroxidation, protect mitochondrial function, and thereby reduce granulosa cell death. GPX4 activators such as selenium supplements can enhance antioxidant capacity (83–86). In addition, nutritional and lifestyle interventions also modulate ferroptosis. N-3 PUFAs, which are usually found in deep-sea fish, nuts, seeds, and leafy greens, regulate the Hippo-YAP pathway, while antioxidants such as vitamin E and coenzyme Q10 inhibit lipid peroxidation. Regular exercise improves iron metabolism and redox homeostasis (85–87). Conventional PCOS treatments may also act through anti-ferroptotic mechanisms. Metformin activates the AMPK/SIRT3 pathway, inhibit ferroptosis, and improve insulin sensitivity and ovarian function. Rosiglitazone, as a PPARγ agonist, upregulates the expression of SLC7A11 and enhances the cellular antioxidant defense capacity (83, 85, 86).

Future research should focus on elucidating the precise mechanisms linking ferroptosis to PCOS, developing ovary-specific ferroptosis-targeting drug delivery systems, validating the efficacy and safety of ferroptosis inhibitors in clinical trials, and exploring ferroptosis-related biomarkers for diagnosis and prognosis. Multidisciplinary efforts may establish ferroptosis-directed therapy as a cornerstone of precision medicine for PCOS.

#### 2.4.7 Ovarian cancer

OC is one of the most lethal malignancies of the female reproductive system worldwide, characterized by high mortality, frequent recurrence, and chemotherapy resistance. According to the WHO Classification of Female Reproductive Tract Tumors, ovarian cancers are categorized into epithelial ovarian cancer (accounting for 90%), ovarian germ cell tumors, and ovarian sex cord-stromal tumors. Epithelial ovarian cancer has the poorest prognosis, with about 70% of patients diagnosed at an advanced stage (III or IV) and a five-year survival rate of only 45%. Standard treatment involves cytoreductive surgery combined with platinum-paclitaxel chemotherapy; however, most patients eventually develop chemoresistance, leading to treatment failure (90–92).

The tumor microenvironment of ovarian cancer is typically characterized by chronic inflammation and immunosuppression. SARS-CoV-2 infection may further intensify these features, potentially promoting ferroptosis through inflammatory mediators such as IL-6. However, cancer cells have evolved corresponding resistance mechanisms. For instance, activation of the FZD7-β-catenin pathway is frequently observed in ovarian cancer tissue. This pathway not only serves as an oncogenic signal but also represents a crucial mechanism for resisting ferroptosis by upregulating genes like GPX4. Consequently, targeting this pathway could resensitize cancer cells to ferroptosis. It is crucial to clarify that the association between SARS-CoV-2 infection and ovarian cancer is indirect, likely mediated through the modulation of inflammatory and immune states within the tumor microenvironment, thereby influencing disease progression. The ferroptosis mechanisms discussed in this section are primarily presented as a promising therapeutic strategy for ovarian cancer, rather than as a direct pathogenic cause initiated by the virus.

Numerous studies have indicated that ferroptosis plays a significant role in the pathogenesis and progression of OC through multiple signaling pathways. Among these, the transcription factor Nrf2 is a key regulatory conferring resistance to ferroptosis, primarily by modulating iron metabolic homeostasis via the HERC2–VAMP8–NCOA4 axis. Specifically, HERC2, a direct transcriptional target of Nrf2, is downregulated upon Nrf2 deficiency. Under normal conditions, HERC2 promotes the ubiquitin-mediated degradation of FBXL5 and NCOA4. Reduction in HERC2 thus enhances the stability of both proteins. On one hand, FBXL5 acts as a negative regulator of ferritin synthesis. Decreased IRP2 levels alleviate translational inhibition of ferritin mRNA, thereby ferritin production. On the other hand, NCOA4 overexpression facilitates the recruitment of iron-loaded ferritin to autophagosomes, resulting in excessive iron release and elevated intracellular free iron, ultimately inducing ferroptosis. Furthermore, Nrf2 loss leads to sustained mTOR activation, which promotes TFEB phosphorylation and cytoplasmic retention. Phosphorylated TFEB is unable to initiate lysosomal gene transcription, and expression of its downstream target VAMP8—a SNARE complex protein essential for autophagosome-lysosome fusion—is suppressed. This impairs autophagosome-lysosome fusion and blocks ferritin degradation, collectively contributing to iron accumulation and ferroptosis (93).

Additionally, Frizzled-7 (FZD7) has been identified as a biomarker in platinum-resistant OC cells and participates in ferroptosis regulation through the FZD7–β-catenin–Tp63–GPX4 signaling axis. FZD7 activation promotes β-catenin/Tp63 signal transduction, thereby upregulating the expression of multiple glutathione metabolism-related genes (such as GSS, GCLC, and SLC7A11), which in turn enhances GPX4 expression. Inhibition of FZD7 reduces GPX4 levels and sensitizes OC cells to ferroptosis (92).

Additional key regulators contribute to the regulation of ferroptosis in OC. Stearoyl-CoA desaturase-1 (SCD1) is highly expressed in OC cells, and its inhibition or knockdown induces ferroptosis. Combining SCD1 inhibitors with ferroptosis inducers significantly enhances antitumor efficacy. Meanwhile, miR-1-3p increases the sensitivity of OC cells to ferroptosis inducers by targeting and reducing FZD7 expression. PARP inhibitors downregulate SLC7A11 in a p53-dependent manner, reducing GSH biosynthesis and promoting lipid peroxidation and ferroptosis. The combination of olaparib and arsenic trioxide activates the AMPKα pathway and suppresses SCD1 expression, synergistically inducing ferroptosis (92).

Combining ferroptosis-targeting strategies with conventional therapeutic can improve OC treatment efficacy. Combining PARP inhibitors and ferroptosis inducers demonstrates a synergistic effect in BRCA wild-type OC, while sodium citrate enhances carboplatin sensitivity. Cisplatin can also promote ferroptosis and improve therapeutic response. In the context of immunotherapy, ferroptosis induces the release of DAMPs, such as HMGB1 and ATP, which activate immune responses and enhance the efficacy of PD-1/PD-L1 inhibitors, thereby modulating the tumor immune microenvironment (90–92, 94).

In summary, ferroptosis demonstrates considerable potential in the treatment of OC. Modulation of the FZD7–β-catenin–TP63 pathway and the Nrf2–HERC2/VAMP8–NCOA4 network can effectively induce ferroptosis in OC cells (92, 93). Particularly in platinum-resistant OC, the combination of ferroptosis inducers with existing therapies—such as PARP inhibitors and immunotherapy—holds significant clinical value (90, 92). Future research should focus on developing more specific ferroptosis inducers, elucidating the crosstalk between ferroptosis and the tumor microenvironment, establishing predictive biomarker systems based on ferroptosis-related mechanisms, and further optimizing combination treatment regimens and dosing strategies. Advancing our understanding of ferroptosis may provide novel therapeutic opportunities for ovarian cancer patients, especially those with resistant disease.

#### 2.4.8 Spontaneous abortion

SA, defined as the spontaneous termination of a pregnancy in the absence of medical intervention, arises from various factors related to the fetus, the mother, or external causes (95). Epidemiological data indicate that SA occurs in approximately 2% of all clinical pregnancies (96). SARS-CoV-2 infection is a significant risk factor for spontaneous abortion, potentially by accelerating the process of placental ferroptosis. Existing evidence, particularly from genetic intervention models, supports a causal role for the involved pathways.

Emerging evidence suggests that SARS-CoV-2 infection may exacerbate the ferroptosis process through several molecular pathways, thereby contributing to SA pathogenesis. One proposed mechanism involves the activation of the HMGB1/ACSL4 axis. Clinical evidence has indicated the significant upregulation of both HMGB1 and ACSL4 in placental villous tissues from SA patients, with expression levels showing a positive correlation. Viral components, such as LPS-like inflammatory factors, can promote HMGB1 expression, which in turn binds to and stabilizes ACSL4 protein. This interaction promotes lipid peroxidation, as evidenced by increased MDA and decreased GSH levels, along with iron accumulation, ultimately leading to trophoblast cell death (97).

Another pathway involves hypoxia-induced signaling within the placental microenvironment following SARS-CoV-2 infection. Under hypoxic conditions, SUMOylated HIF1α (HIF1α-SUMO) directly activates NCOA4, triggering ferritin degradation and the release of labile iron. The long non-coding RNA lnc-HZ06 maintains the stability of HIF1α-SUMO by inhibiting the deSUMOylating enzyme SENP1, thereby forming a positive feedback loop that enhances the ferroptosis process. This mechanism has been corroborated in both human SA villous tissues and hypoxic mouse models, where knockdown of lnc-HZ06 reduced the miscarriage rate (98).

Furthermore, the RNA-binding protein IGF2BP3 may contribute to recurrent pregnancy loss by stabilizing the expression of ferroptosis-related mRNAs such as TFR1 (99), further promoting iron uptake and cellular vulnerability to ferroptotic death.

The aforementioned pathways may interact with oxidative stress responses triggered by SARS-CoV-2, creating a synergistic effect that induce ferroptosis in trophoblast and/or endothelial cells. Key mechanisms include HMGB1/ACSL4 pathway, the hypoxia/HIF1α-SUMO axis, and GPX4 degradation. Therapeutic strategies targeting these pathways, such as HMGB1 inhibitors or lnc-HZ06 antagonists, could offer novel avenues for the treatment of SA.

#### 2.4.9 Endometriosis

EMs is a chronic gynecological disorder characterized by the presence of endometrial-like tissue outside the uterus cavity, affecting 5 - 10% of women of reproductive age globally (100). Emerging evidence indicates that SARS-CoV-2 infection exacerbates ferroptosis in endometriosis through multiple interconnected molecular mechanisms. It should be noted that the discussion here centers on the exacerbating effect of viral infection on a preexisting condition, for which the evidence is largely based on plausible inferences from mechanistic correlations. Following viral entry, downregulation of the ACE2 receptor leads to accumulation of angiotensin II, which activates NADPH oxidase and enhances the generation of ROS. Simultaneously, the virus-induced cytokine storm (e.g., TNF-α, IL-6) synergizes with the chronic inflammatory microenvironment typical of EMs, further suppressing GPX4 activity and upregulating TFRC expression to increase cellular iron uptake.

Iron levels are significantly elevated in the peritoneal and follicular fluids of EMs patients. This iron excess facilitates ROS production via the Fenton reaction, inducing lipid peroxidation damage and subsequent cellular damage. Moreover, NCO4-mediated ferritinophagy is enhanced, leading to further releases of free iron ions and amplification of oxidative stress. Viral proteins may also synergize with ferroptosis to disrupt mitochondrial integrity, resulting in reduced ATP synthesis and cellular dysfunction.

Notably, EMs cells develop resistance mechanisms to resist ferroptosis by upregulating key factors such as SLC7A11 and GPX4, thereby promoting disease persistence and progression. Therapeutically, targeting ferroptosis with iron chelators (e.g., deferoxamine) or antioxidants (e.g., vitamin E) may may offer promising strategies to attenuate iron dysmetabolism and oxidative damage, thereby potentially alleviating EMs-related symptoms (101, 102). The commonalities and specificities of ferroptosis mechanisms in SARS-CoV-2-Related reproductive system diseases are shown in **Table 2**.

**Table 2:** Commonalities and specificities of ferroptosis mechanisms in SARS-CoV-2-related reproductive system diseases.

Disease	Common upstream pathway: role of the IL-6/NF-κB inflammation-ferroptosis cycle	Disease-specific key targets/pathways	Basis of tissue-intrinsic susceptibility
Male Infertility/Orchitis	Viral-triggered IL-6 release disrupts systemic and testicular iron homeostasis, establishing a foundation for ferroptosis.	Plin4: Specifically expressed in testicular Leydig cells; its dysregulation directly leads to aberrant lipid metabolism and mitochondrial dysfunction.	The testicular spermatogenic environment requires immune privilege, which, when disrupted by the virus, results in intense inflammation. Spermatogenic cell membranes are rich in polyunsaturated fatty acids (PUFAs).
Preeclampsia/Eclampsia (PE)	Systemic inflammatory response exacerbates placental oxidative stress, acting synergistically with physiological placental hypoxia-reperfusion injury.	MLL1/RBM15 Epigenetic Axis: Specifically regulates the mRNA stability of TRIM72, a key ferroptosis-related protein in the placenta.	Placental trophoblast cells exhibit highly active iron metabolism to meet fetal demands, rendering them particularly sensitive to iron overload and oxidative damage.
Ovarian Cancer (OC)	Infection may intensify inflammation in the tumor microenvironment, suppress anti-tumor immunity, and simultaneously create conditions that promote ferroptosis.	FZD7/β-catenin/TP63 Axis: Specifically activated in cancer cells, conferring resistance to ferroptosis by regulating genes involved in glutathione (GSH) metabolism.	The rapid proliferation of ovarian epithelial cancer cells results in heightened metabolic activity, increasing their sensitivity to alterations in iron and lipid metabolism.
Polycystic Ovary Syndrome (PCOS)	Viral infection may amplify the pre-existing chronic low-grade inflammatory state in PCOS, thereby accelerating the ferroptosis process.	Hyperandrogenism-Ferroptosis Cycle: Dihydrotestosterone (DHT) upregulates factors like NCOA4, forming a self-amplifying pathological loop unique to PCOS.	Ovarian granulosa cell function is highly dependent on precise energy metabolism and redox homeostasis, making them susceptible to metabolic dysregulation.
Endometriosis (EMs)	Virus-induced inflammation interacts with the intrinsic chronic inflammatory microenvironment of EMs lesions, forming a vicious cycle.	ACE2 Downregulation-Ang II-ROS Axis: In EMs tissues, the virus may specifically exacerbate oxidative stress via this pathway.	Ectopic endometrial lesions exist in a microenvironment of relative ischemia, hypoxia, and recurrent hemorrhage, creating a propensity for iron accumulation.

### 2.5 Ferroptosis and other reproductive tract infection-associated pathogens

*Chlamydia trachomatis* (*C. trachomatis*) is a common sexually transmitted pathogen that can cause a range of diseases including trachoma, urethritis, cervicitis, pelvic inflammatory disease, tubal factor infertility, and ectopic pregnancy. Recent studies have revealed that *C. trachomatis* can actively regulate the ferroptosis process in host cells, a mechanism that plays a key role in its pathogenesis. Infection with *C. trachomatis* can induce ferroptosis in host cells. Infected cells display typical features of ferroptosis, including increased iron content, accumulation of ROS, lipid peroxidation, and mitochondrial damage. Transcriptomic analysis shows significant alterations in the expression of ferroptosis-related genes in *C. trachomatis*-infected host cells, including upregulation of genes involved in iron metabolism, lipid metabolism, and oxidative stress (e.g., FTL, ACOX1, SAT1, HMOX1), and downregulation of negative regulators of ferroptosis (e.g., GPX2, SLC7A11, SLC1A4) (103). Notably, the ferroptosis induced by *C. trachomatis* appears to facilitate its own replication. Using the ferroptosis-specific inhibitor Fer-1 significantly suppresses *C. trachomatis* replication, whereas the ferroptosis inducer Erastin enhances its replicative ability (103). As an obligate intracellular pathogen, *C. trachomatis* cannot grow or replicate independently outside host cells and must rely on the host cell’s energy and nutrient systems to complete its unique life cycle. At the late stage of infection, *C. trachomatis* promotes the release of progeny elementary bodies by causing host cell lysis. Research indicates that *C. trachomatis* promotes the release of progeny chlamydiae by inducing ferroptosis in the host cell. Treatment with Vitamin E (a ferroptosis inhibitor) markedly suppressed the release of progeny elementary bodies (104). Mechanistically, firstly, *C. trachomatis*, via its own CPAF protein, degrades the host cell’s SLC7A11 protein, leading to intracellular SLC7A11 depletion and inhibition of GPX4 synthesis, thereby promoting ferroptosis. Secondly, *C. trachomatis* enhances the expression of p53 in host cells, which in turn suppresses SLC7A11 transcription, reduces its protein levels, and ultimately promotes ferroptosis (104, 105).

*Candida albicans* (*C. albicans*) is a common commensal fungus that typically colonizes mucosal sites such as the oral cavity, intestines, and urogenital tract. When the host’s immune balance is disrupted or the local microenvironment changes, it can over-proliferate and transition to a pathogenic state, causing endogenous infections (e.g., candidal vulvovaginitis, candidal balanitis). Studies show that the key virulence factor CVF1 of *C. albicans* plays an important role in its pathogenicity. CVF1 can specifically induce ferroptosis in invading macrophages, characterized by depletion of intracellular antioxidants (e.g., GSH, GPX4) and significant accumulation of lipid peroxides (e.g., 4-HNE, MDA). After macrophages undergo ferroptosis, not only is their phagocytic and fungicidal capacity impaired, allowing *C. albicans* to survive and disseminate, but they also release inflammatory factors, triggering a strong inflammatory response and exacerbating tissue damage. Research confirms that treatment with the ferroptosis-specific inhibitor Fer-1 effectively mitigates CVF1-mediated fungal dissemination, tissue damage, and excessive inflammation (106).

*Pseudomonas aeruginosa* (*P. aeruginosa*), a Gram-negative bacillus, is a common nosocomial pathogen. It can cause urogenital tract infections (e.g., balanitis, vulvovaginitis, and necrotizing fasciitis of the perineum) due to improper medical procedures or immunocompromised states. *P. aeruginosa* utilizes the quorum-sensing signal molecule PQS to epigenetically regulate iron metabolism in macrophages, amplifying iron uptake and inducing ferroptosis. The molecular mechanism involves the PQS-CNMT-TFR1 axis: upon activation by PQS, CNMT catalyzes the methylation of histidine at position 35 in the cytoplasmic domain of the transferrin receptor 1 (TFR1) protein on the cell membrane. This enhances the cell’s iron uptake capacity, leading to overload of the intracellular labile iron pool (LIP). The cell death induced by PQS can be rescued using the ferroptosis inhibitor Fer-1 (107).

Multiple pathogens associated with reproductive system infections have been reported to exhibit features of ferroptosis during infection, such as dysregulated iron metabolism, lipid peroxidation, and mitochondrial damage, indicating that ferroptosis likely plays a crucial role in their pathogenicity. However, due to insufficient research, the specific regulatory mechanisms of ferroptosis in these contexts remain incompletely understood, which significantly impedes the development of effective therapeutic strategies targeting this pathway. Therefore, elucidating the regulatory mechanisms of ferroptosis and exploring it as an interventional target are of great importance for deepening our understanding of disease progression and developing more effective treatments. The interaction between pathogens and ferroptosis in reproductive system infections are summarized in **Table 3**.

**Table 3:** Interaction between pathogens and ferroptosis in reproductive system infections: key targets, mechanisms, and therapeutic potential.

Disease/pathogen	Key target(s)	Candidate modulator(s)	Mechanism of action	Study model	Therapeutic potential
HIV (INRs)	GPX4, Nrf2	Ferrostatin-1, Luteolin	Inhibits ferroptosis in CD4+ T cells and improves mitochondrial function.	*In vitro*T-cell model	Promotes immune reconstitution and reduces inflammation.
HPV-associated Cervical Cancer	SLC7A11, GPX4	HOXA5 overexpression, Erastin	Downregulates SLC7A11 to induce ferroptosis in tumor cells.	Cell line/Mouse model	Enhances chemosensitivity and suppresses tumor growth.
*S. aureus*-induced Endometritis	Nrf2/GPX4 pathway	Luteolin, Caffeic Acid	Activates the Nrf2/GPX4 pathway, suppressing both inflammation and ferroptosis.	Mouse endometritis model	Alleviates tissue damage and inhibits inflammatory responses.
*S. aureus*-induced Mastitis	SLC7A11, GPX4	Cyanidin-3-O-glucoside	Activates SESN2/Nrf2 signaling, inhibiting ferroptosis and NF-κB pathway activation.	Mouse mastitis model	Mitigates mammary gland inflammation and oxidative damage.
SARS-CoV-2-associated Male Infertility	GPX4, Nrf2	Ferrostatin-1, Ursolic Acid	Suppresses ferroptosis in testicular cells and improves sperm quality.	Mouse model/Clinical observations	Improves sperm parameters and restores blood-testis barrier function.
Preeclampsia/Eclampsia​	SLC7A11, GPX4	miR-30b-5p inhibitor	Upregulates SLC7A11 and PAX3 expression, inhibiting ferroptosis in placental trophoblasts.	Human placental tissue/Mouse model	Ameliorates hypertension and proteinuria, improving pregnancy outcomes.
Ovarian Cancer	FZD7, Nrf2, SCD1	PARP inhibitor + Erastin	Downregulates SLC7A11 and induces lipid peroxidation.	Cell line/Mouse model	Overcomes platinum resistance and enhances efficacy of immunotherapy.

### 2.6 Common mechanisms of ferroptosis across different pathogens

Although HIV, HPV, *S. aureus*, and SARS-CoV-2 employ distinct mechanisms to infect the reproductive system, ferroptosis emerges as a highly conserved core signaling pathway in their pathological processes.

**Figure 4:**
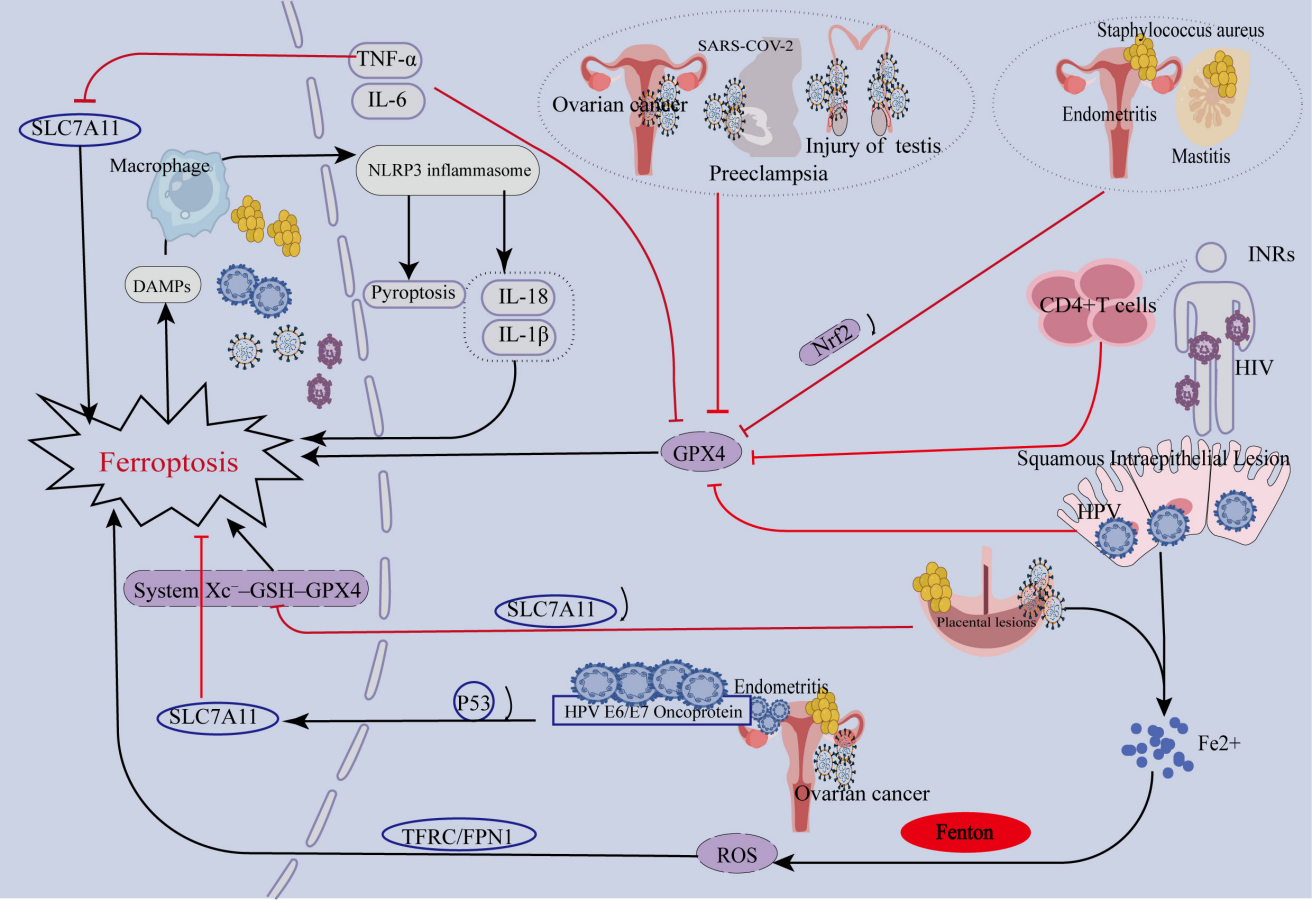
Regulatory network of different pathogens on host ferroptosis mechanisms. The figure illustrates the multi-level regulation of host iron homeostasis and the ferroptosis pathway by HPV, HIV, SARS-CoV-2, and *Staphylococcus aureus* infections. Ferroptotic cells release DAMPs, activating macrophages and the NLRP3 inflammasome, which induces IL-1β and IL-18 production and promotes pyroptosis, thereby amplifying inflammatory responses. Concurrently, inflammatory signals can exacerbate iron homeostasis disruption, creating a mutual reinforcement between ferroptosis and inflammation. The System Xc^−^–GSH–GPX4 axis serves as the core inhibitory pathway of ferroptosis, with GPX4 regulated by Nrf2 and SLC7A11 responsible for extracellular cystine uptake to maintain GSH synthesis. In *S. aureus* infection and SARS-CoV-2-associated placental lesions and male infertility, downregulation of SLC7A11 leads to GSH depletion and failure of antioxidant defense. HPV E6/E7 upregulates SLC7A11 by degrading p53, a key mechanism for acquiring ferroptosis resistance during later stages of carcinogenesis. The overall mechanism is closely linked to various reproductive tract infections and tissue damage. All the figures are created in https://BioRender.com.

First, dysregulation of the Nrf2/GPX4 signaling axis is a hallmark event in infections caused by multiple pathogens. The downregulation of GPX4 expression and loss of its activity, as a crucial negative regulator of ferroptosis, is a key step in triggering ferroptosis. GPX4 expression is significantly downregulated in CD4^+^ T cells of NRs. Its activity is suppressed during HPV-associated SIL stages; its expression and function are inhibited in *S. aureus-*induced endometritis and mastitis; and GPX4 depletion is a central event triggering ferroptosis in tissue cells in SARS-CoV-2-associated testicular damage, pre-eclampsia, and ovarian cancer. As a key upstream transcriptional regulator of GPX4, the inactivation of Nrf2 (e.g., via IFP35-mediated degradation during *S. aureus* infection) or its compensatory activation (e.g., in some tumor cells) collectively determines cellular susceptibility to ferroptosis (**Figure 4**).

Second, inhibition of the System Xc^−^–GSH–GPX4 signaling pathway represents another common route for ferroptosis induction. SLC7A11, the core component of System Xc^−^, transports extracellular cystine into the cell for GSH synthesis. It plays a critical role in HPV persistent infection, *S. aureus*-induced endometrial inflammation, and SARS-CoV-2-associated ovarian cancer and pregnancy complications (**Figure 4**). HPV E6/E7 oncoproteins indirectly promote SLC7A11 expression by degrading p53, representing a mechanism for acquiring ferroptosis resistance during later stages of carcinogenesis. Conversely, in *S. aureus* infection and SARS-CoV-2-related placental pathologies and male infertility, SLC7A11 expression is significantly suppressed, leading to GSH depletion and collapse of the antioxidant defense.

Third, disruption of iron metabolism catalyzes ferroptosis. Excessive accumulation of intracellular Fe²^+^ triggers a burst of ROS production via the Fenton reaction, which directly attacks polyunsaturated fatty acids, initiating lipid peroxidation. This phenomenon is widely observed in HPV-mediated cervical intraepithelial neoplasia, ferroptosis in PCOS ovarian granulosa cells, and COVID-19-associated placental iron overload, further fueling the Fenton reaction and promoting lipid peroxidation. The molecular basis typically involves increased iron uptake mediated by TFRC upregulation and/or impaired iron efflux due to FPN1 downregulation.

More importantly, a “ferroptosis-inflammation positive feedback loop” is prevalent across many reproductive tract infections. Pathogen infection or viral proteins first induce a chronic inflammatory microenvironment. Released inflammatory cytokines, such as TNF-α and IL-6, can suppress the expression of key antioxidant proteins like GPX4 and SLC7A11, thereby initiating ferroptosis. Subsequently, host cells undergoing ferroptosis release large quantities of DAMPs, such as HMGB1 and ATP. These DAMPs act as danger signals, recognized by pattern recognition receptors on immune cells like macrophages, leading to activation of the NLRP3 inflammasome. NLRP3 inflammasome activation not only results in the maturation and release of pro-inflammatory cytokines IL-1β and IL-18 but also cleaves GSDMD, inducing pyroptosis, which further amplifies the inflammatory signal. Thus, a vicious cycle is formed: “inflammation → ferroptosis → DAMP release → NLRP3 activation → stronger inflammation.” This cycle impedes immune reconstitution in HIV infection, exacerbates suppurative tissue damage in *S. aureus* infection, promotes multi-organ dysfunction in SARS-CoV-2 infection, and shapes a chronic inflammatory microenvironment favorable for tumor growth in HPV-associated carcinogenesis (**Figure 4**).

Investigating these common signaling pathways not only deepens our understanding of the pathological mechanisms underlying reproductive tract infections but also provides a theoretical foundation for developing broad-spectrum or precisely targeted ferroptosis-based therapeutic strategies.

## 3 Interrelationships among ferroptosis, apoptosis, and pyroptosis in reproductive tract infections and dysfunction

In infectious diseases of the reproductive system and related reproductive disorders, ferroptosis does not act in isolation but interacts with both apoptosis and pyroptosis. Their interplay collectively determines whether the outcome of an infection is an appropriate immune response or excessive immune-mediated damage.

### 3.1 Interaction between ferroptosis and pyroptosis triggers a cytokine storm

In human reproductive system infections and related disorders, a positive feedback loop exists between ferroptosis and pyroptosis, which is a key mechanism driving the vicious cycle of infectious inflammation. Following membrane lipid peroxidation and rupture, ferroptotic cells release large amounts of DAMPs, such as HMGB1 and ATP. These endogenous danger signals can be recognized by pattern recognition receptors (e.g., TLR4) on immune cells like macrophages, leading to potent activation of the NLRP3 inflammasome. The activated NLRP3 inflammasome recruits and activates caspase-1 via the adaptor protein ASC. On one hand, active caspase-1 cleaves the precursors of IL-1β and IL-18, generating mature, strongly pro-inflammatory cytokines. On the other hand, it cleaves the GSDMD protein, enabling its N-terminal domains to oligomerize and form pores in the plasma membrane, causing cell swelling, rupture, and release of cellular contents—a form of inflammatory cell death known as pyroptosis. The additional inflammatory cytokines (including IL-1β) and DAMPs released by pyroptotic cells can further act on surrounding cells, inducing more ferroptosis and pyroptosis, thereby establishing a self-amplifying circuit: “Ferroptosis → DAMP release → NLRP3 activation/Pyroptosis→ Cytokine storm → More ferroptosis.” This mechanism has been experimentally confirmed in *S. aureus*-induced endometritis and mastitis, as well as in SARS-CoV-2-associated orchitis and systemic inflammatory responses, representing a significant cause of tissue destruction and disease severity (44, 108, 109).

### 3.2 The apoptotic signaling pathway regulates ferroptosis

Ferroptosis and apoptosis share common upstream signaling molecules, with p53 being the most representative. In HPV-associated cervical carcinogenesis, E6 oncoprotein-mediated degradation of p53 relieves its transcriptional repression of SLC7A11, leading to upregulated SLC7A11 expression and enhanced GSH synthesis. This is a key mechanism by which cervical cancer cells acquire ferroptosis resistance and enable malignant progression (20). Conversely, in *S. aureus* infection, stably expressed p53 can suppress SLC7A11 transcription, thereby promoting ferroptosis (110). Furthermore, classical apoptosis regulators like BCL-2 have been found to indirectly modulate cellular susceptibility to ferroptosis by influencing the mitochondrial voltage-dependent anion channel (VDAC) or directly interacting with proteins like GPX4, revealing interactions between these two death pathways at the organelle level.

### 3.3 The interplay among the three determines infection outcomes

During different stages and in various cell types involved in reproductive tract infections and related disorders, these three cell death modalities exhibit dynamic synergistic or antagonistic relationships. Synergistic interactions are commonly observed in early host defense. For instance, during the SIL stage of HPV infection, ferroptosis is utilized to efficiently clear virus-transformed epithelial cells, while concurrent apoptosis also contributes, jointly controlling the infection. However, in chronic or late-stage infections, the synergy between ferroptosis and pyroptosis primarily inflicts immune damage on the host. Particularly under conditions of immunosuppression (e.g., HIV infection) or imbalanced tissue microenvironments (e.g., pre-eclamptic placenta), this synergy can lead to uncontrollable cytokine storms and tissue necrosis. Antagonism among ferroptosis, apoptosis, and pyroptosis also occurs and influences disease progression. A prime example is HPV-driven cervical SCC (squamous cell carcinoma), where cancer cells upregulate anti-ferroptosis pathways through mechanisms such as the aforementioned p53 degradation and Nrf2 activation, enabling them to survive under pressures that would normally trigger ferroptotic clearance. In this scenario, the cancer cells’ resistance to ferroptosis antagonizes their retained susceptibility to apoptosis, but the overall advantage shifts towards survival and proliferation.

In summary, ferroptosis, apoptosis, and pyroptosis constitute a sophisticated and dynamic cell death network. Their interactions in the context of reproductive tract infections and related disorders influence the entire process, from pathogen clearance and inflammatory responses to tissue repair and fibrosis. Understanding the interplay among these three programmed cell death modalities is not only crucial for a complete understanding of the pathophysiology of infectious diseases but also provides a novel perspective and theoretical foundation for designing and developing combined therapeutic strategies targeting “anti-infection — ferroptosis — apoptosis — pyroptosis.”

## 4 Conclusion

This review systematically elaborates on the pivotal role of ferroptosis in human reproductive system infections and associated reproductive disorders, covering a range of representative pathogens including HIV, HPV, *S. aureus*, SARS-CoV-2, *Chlamydia trachomatis*, and *Candida albicans*. By integrating current research, we demonstrate that ferroptosis serves as a core mechanism mediating host tissue damage induced by pathogenic infections. Ferroptosis exhibits a complex double-edged sword characteristic in these contexts: during early infection or pre-cancerous stages, it can function as a host defense mechanism to eliminate pathogens and abnormal cells; whereas in chronic infections or advanced disease stages, uncontrolled ferroptosis exacerbates tissue damage, inflammatory storms, and immune dysfunction, thereby driving disease progression towards malignancy.

Our analysis reveals that despite the diversity of pathogens, they commonly disrupt conserved core pathways of ferroptosis in the host. Dysregulation of the Nrf2/GPX4 antioxidant defense system, disruption of the System Xc^−^–GSH synthesis axis, and imbalance in iron metabolism homeostasis constitute shared mechanisms triggering ferroptosis across different infectious backgrounds. Crucially, we identify the widespread presence of a “ferroptosis-inflammation positive feedback loop” across various pathogen infections. Pathogen infection or viral proteins induce the release of inflammatory cytokines such as IL-6 and TNF-α, which subsequently suppress key antioxidant proteins like GPX4 and SLC7A11, initiating ferroptosis. In turn, DAMPs like HMGB1 released by ferroptotic cells activate the NLRP3 inflammasome, intensifying the cytokine storm and promoting pyroptosis, thereby forming a self-amplifying vicious cycle. This cycle is evident in HIV-associated INRs, suppurative injuries caused by *S. aureus*, and multi-organ dysfunction resulting from SARS-CoV-2 infection, accelerating the pathological progression of these conditions. Simultaneously, different pathogens also exhibit specific mechanisms during infection. For instance, HIV viral proteins directly interfere with host cell selenium metabolism and organellar iron homeostasis, while HPV confers ferroptosis resistance to cells in late-stage cervical carcinogenesis by degrading p53 and activating oncogenic pathways. The coexistence of these common pathways and pathogen-specific mechanisms provides a theoretical basis for developing therapeutic strategies that combine broad-spectrum and precision-targeting approaches.

We further discovered that ferroptosis does not operate in isolation within the cell death network but rather forms an interactive network with apoptosis and pyroptosis. Their relationships involve both synergy and antagonism. During the SIL stage of HPV infection, ferroptosis and apoptosis cooperate to clear infected cells. In contrast, during *S. aureus* infections and severe SARS-CoV-2 complications, the synergy between ferroptosis and pyroptosis contributes to the inflammatory storm responsible for tissue destruction. Conversely, resistance to ferroptosis (e.g., in HPV-associated cervical cancer) becomes key for pathogens or tumor cells to achieve immune escape and malignant progression. Understanding this complex interactive network is crucial for developing combined treatment strategies tailored to different infection stages and pathological states.

Targeting ferroptosis presents significant potential in the treatment of infectious diseases affecting the human reproductive system. In HIV-infected INRs, ferroptosis inhibitors can improve mitochondrial function in CD4^+^ T cells, attenuate inflammation and metabolic dysregulation, reduce viral replication, and disrupt the erroptosis–inflammation cycle. In HPV-associated cervical cancer, combining ferroptosis inhibitors such as GPX4 with conventional chemotherapy, or employing ferroptosis inducers alongside chemotherapeutic drugs like cisplatin, as well as targeting the KRAS/Nrf2/SLC7A11 signaling axis, may help overcome ferroptosis resistance and restore susceptibility to ferroptosis. Strategies targeting the KRAS/Nrf2/SLC7A11 axis also hold potential to suppress malignant progression. In *S. aureus* infections, luteolin inhibits host cell ferroptosis via the Nrf2/GPX4 pathway, thereby reducing cytotoxicity. Moreover, inducing ferroptosis-like bacterial cell death in *S. aureus* offers a novel strategy to eliminate drug-resistant bacteria, representing a promising alternative strategy to counter multidrug resistance.

Although targeting ferroptosis shows significant therapeutic promise, its clinical translation still faces challenges. Currently, most evidence is still derived from cell and animal models, and the efficacy, tissue specificity, and long-term safety of ferroptosis inhibitors or inducers in humans urgently require validation. Furthermore, given the double-edged sword role of ferroptosis in human reproductive system infections and related disorders, interventional strategies targeting it must be carefully timed. For instance, inducing ferroptosis might be necessary to eliminate pathogens during the early stages of infection, whereas inhibiting ferroptosis may be required to protect tissues during the chronic phase.
